# Surgical Education Within Planetary Health Curricula: A Global Environmental Scan (2022–2025)

**DOI:** 10.3390/ijerph22101545

**Published:** 2025-10-10

**Authors:** Rosemary Vayalikunnel, Poliana Zanotto Manoel, Agnes Zanotto Manoel, Mathanky Jeyakumar, Le Qi Chen, Rushan Jeyakumar, Patricia Balmes, Annie Lalande, Andrea J. MacNeill, Shahrzad Joharifard, Emilie Joos

**Affiliations:** 1 Faculty of Science, University of British Columbia, Vancouver, BC V6T 1Z1, Canada; sc0996t1@student.ubc.ca (L.Q.C.); patricia.balmes@vch.ca (P.B.); 2 Global Surgery Lab, Branch for Global Surgical Care, Department of Surgery, University of British Columbia, Vancouver, BC V5Z 1M9, Canadajeyakr2@mcmaster.ca (R.J.);; 3 Faculty of Medicine, Federal University of Rio Grande, Rio Grande 96200-400, RS, Brazil; 4 Schulich School of Medicine and Dentistry, Western University, London, ON N6A 5C1, Canada; 5 Faculty of Medicine, University of British Columbia, Vancouver, BC V6T 1Z3, Canada; 6 Arts & Science Program, McMaster University, Hamilton, ON L8S 4K1, Canada; 7 Division of General Surgery, Vancouver General Hospital, Vancouver, BC V5Z 1M9, Canada; 8 Planetary Healthcare Lab, Faculty of Medicine, University of British Columbia, Vancouver, BC V6T 1Z3, Canada; 9 Division of Pediatric Surgery, British Columbia Children’s Hospital, Vancouver, BC V6H 3V4, Canada

**Keywords:** planetary health, education, surgery, surgical care, carbon footprint, perioperative care, accessibility, availability

## Abstract

Operating rooms (ORs) represent strategic targets for climate mitigation efforts, given their significant environmental footprint and the need for increased surgical capacity to meet the global surgical burden of disease. OR teams are often unaware of impacts of unsustainable surgical practices. Although research supports the integration of planetary health into clinical education, there is limited data on the availability, structure, and surgical content of such courses. This study examines the availability and accessibility of planetary health courses (PHCs) worldwide, with a focus on identifying surgical content within curricula. An environmental scan was conducted using internet searches, reviewing curricula from the top ten universities in each global region and cross-referencing existing course collections. Courses were evaluated based on type, cost, language, and whether they addressed the environmental impact of surgery. A total of 248 courses were identified, primarily at the graduate level, offered in English, and concentrated in North America and Europe. Only four courses included content on the intersection of planetary health and surgery. These findings demonstrate the lack of surgical content in planetary health education and emphasize the need to develop comprehensive, accessible, and globally representative courses that address the environmental impacts of surgical care.

## 1. Introduction

Planetary health refers to the recognition of the connection between human health and the environmental state of our planet. The relationship is bidirectional: while changes within the environment directly impact human health, healthcare utilization has shown to cause direct changes to the environment [1,2]. Planetary health was first described in 2015 in the Rockefeller Foundation–Lancet Commission on Planetary Health, and since then, studies have continued to demonstrate the far-reaching impacts of climate change and pollution on all aspects of health across all populations [1,3]. The healthcare sector has been identified as an important driver of climate change, being responsible for approximately 4.6% of global greenhouse gas emissions [3]. This has resulted in global efforts, such as the COP26 Health Programme, to accelerate the transition towards sustainable, resilient, and low-carbon healthcare systems [4]. Reducing the GHG emissions of healthcare systems requires transformations at all levels including the local level, from the healthcare providers themselves, such as adopting low-carbon treatments; at the institutional and governmental level, such as developing energy-efficient infrastructure and transitioning to renewable energy sources; and at a global public health level to improve population health and thus decrease the demands on healthcare services [5].

Most of the impacts of health services delivery are attributable to energy and resource utilization, and acute care settings, such as hospitals, are the most environmentally intensive sites of treatment [6,7,8]. Within hospitals, operating theaters have been identified as strategic targets for climate change mitigation [9]. A 2017 study quantified the carbon footprint of operating rooms in Canada, the United States of America, and the United Kingdom and found an average annual footprint of ~188 tCO2 per operating room [9]. Moreover, the global burden of greenhouse gas (GHG) emissions associated with operating rooms is expected to grow considerably due to the increasing demand for surgical care, as it is essential for prevention, treatment, diagnosis, and emergency care. A study estimated that an additional 143.1 million surgical procedures would be needed yearly to reach the 321.5 million surgical procedures needed globally in 2010 [10], and this number continues to rise with population growth and increasing healthcare needs. As surgical capacity and workforce expand worldwide, it becomes increasingly critical to integrate surgical education into planetary health resources to equip surgical teams with the knowledge and skills to deliver high-quality, low-carbon, sustainable care. Understanding the environmental impacts of care pathways can help guide decisions between clinically equivalent treatment options and strengthen the case for preventive measures that reduce healthcare burdens and associated emissions [5]. The Planetary Health Education Framework has been brought forth as a tool to guide the development of educational resources, integrating health, social, and environmental education interconnections [11]. With this knowledge, surgical providers can also contribute to developing sustainable and resilient health systems [12], advocate for environmentally responsible practices and improve care for vulnerable populations that are disproportionally affected by climate change [13].

Three main contributors of GHG have been identified within operating rooms: energy use, primarily for heating, ventilation, and air conditioning, anesthetic gases, and consumables [9]. Healthcare professionals can have a direct impact on all three domains. They can reduce the use of the operating room, by offering procedures in an ambulatory setting, as it is more widely carried out with hand surgery [14]. This can increase timely patient access to care while reducing healthcare costs, environmental impacts, and benefiting patient recovery. In terms of anesthesia, many studies have shown that educating the anesthesia workforce on the carbon footprint of different agents can have a direct impact on the emissions within their healthcare system by shifting anesthetic practices [15]. Finally, operating rooms are major contributors to consumable utilization [16], and healthcare providers play an important role in instrument selection for surgical procedures [17]. It is estimated that reducing the utilization of single-use devices would contribute to a decrease of more than 50% of the waste produced by operating rooms, while also improving resilience to supply chain disruptions [9,17,18,19].

As planetary health emerges as a critical component of global health and medical education, there is an increase in the development of related courses in educational and professional settings, which has been well described in a recent scoping review by Asaduzzaman et al. in 2022 [20]. While the 43 planetary health courses identified covered important aspects of the interconnectedness of human and planetary health, none included content around the intersection of surgical care and planetary health, highlighting the evidence to practice gap. While an understanding of the overarching principles of planetary health is important for healthcare practitioners, the integration of field-specific content is essential to enable actionable, context-relevant change. Such targeted guidance empowers clinicians—who may have limited influence over broader public health policy—to enact meaningful improvements through their daily clinical practice. Building on the 2022 scoping review, we therefore widened the search window to 2022–2025, incorporating institutional catalogues and hand-curated course lists, and quantified perioperative and surgical content specifically. Moreover, our study aimed to determine whether PHCs are present in countries that are major contributors to pollution as well as in those most vulnerable to the impacts of climate change. Countries facing extreme weather events need environmental literacy courses tailored to their specific environmental, economic, and cultural contexts to inform effective responses to climate-related issues [21,22].

## 2. Materials and Methods

An environmental scan was conducted using three data sources to identify PHCs (Appendix B.1, Figure A2). A course was defined as any standalone structured educational activity with learning objectives, organized content and/or an evaluation process or credits/certificate upon completion. Planetary health content included any material related to the following keywords “environment”, “climate change”, and/or “sustainable” associated with the term “health”. Surgical content was defined as any mention of “surgery”, “surgical care”, and/or “operating room/theater” within the courses’ syllabus, description or learning objectives. Data collection occurred between 1 December 2022 and 31 May 2025, and the environmental scan provides an overview of course availability in mid-2025. See Appendix B.2 for course inclusion and exclusion criteria.

A Google search for “planetary health courses” was conducted in December 2022, with the first ten pages of results analyzed to compile an initial list of relevant courses (see Appendix B.3). Google was the chosen search platform to mirror the tool that trainees and surgical staff would use when seeking planetary health courses, and the selected keywords reflect the search strategies they would employ. This approach also allowed the capture of course offerings across disciplines, regions and organizations, including smaller programs or certificates not included in academic databases and offered outside of formal educational institutions. Next, we searched for publicly available planetary health curricula on the websites of the top ten universities in each of the six global regions (Africa, Asia, Oceania, Europe, South America, and North America), as ranked by the US News 2022–2023 University rankings [23]. If course information was not publicly available, universities were contacted directly through email to request additional details. The course list was then cross-referenced with existing course inventories created by Asaduzzaman et al. [20] and The Planetary Health Alliance [24]. The school-specific Planetary Health Report Card documents from medical schools [25] were also reviewed, and the identified courses were verified by searching specifically for medical school electives at the institution.

The following data were collected for each course: country of university/organization, type (undergraduate, graduate, certificate, open access, and professional development), duration, cost, language of delivery and inclusion of surgical content. Countries’ contribution to GHG emissions or climate change vulnerability was also captured. Data was collected on the countries’ ranking on the top ten list of contributors of pollutants according to the Carbon Brief site [26], which ranks countries based on the accumulation of CO_2_ emissions from 1850 to 2021, or top ten most vulnerable to the impacts of climate change according to the International Rescue Committee [27], which analyzes countries’ levels of climate readiness and fragility.

Course duration was based on the total duration of study necessary for students to complete the course. Individual undergraduate or graduate courses offered on a semester basis were recorded as lasting one semester. For courses offered in the context of a specific program, including medical school, the duration of the entire program was considered.

The costs were reported in USD and reflected only tuition fees, excluding any additional charges. Like course duration, course cost reflected the total duration of study necessary to complete the course. If the course was an undergraduate or graduate course, the cost of one semester was obtained from the university website. If it was an undergraduate, graduate or medical school program, then the cost for the entire program across the entire duration was considered. When different course fees (for example international versus domestic students) were advertised, we reported the highest course fee.

Courses were collected, included or excluded based on criteria and independently reviewed by multiple authors (see Appendix B.4), and discrepancies were resolved by consensus.

## 3. Results

A total of 248 courses were compiled. The full results are available in Appendix A and Supplementary Materials.

### 3.1. Geographic Distribution of Courses

**Figure A1:**
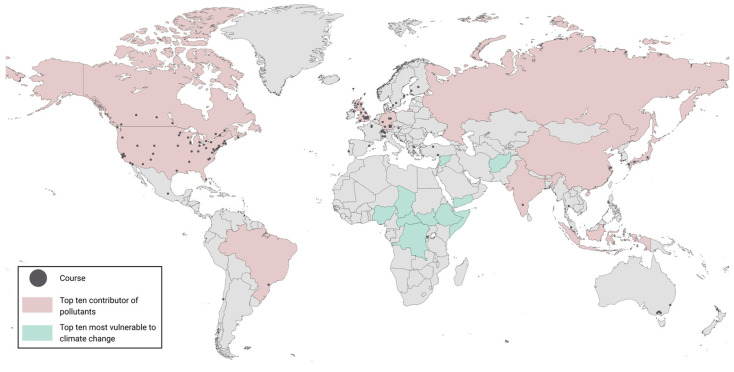
Geographical Distribution of Courses.

**Figure A2:**
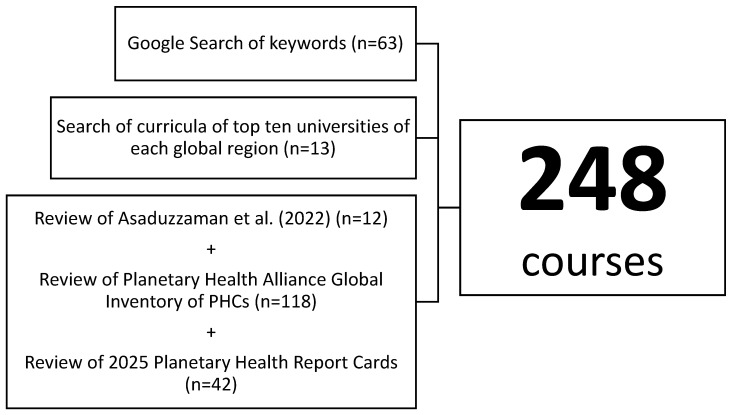
Number of courses compiled from 3 data collection methods.

PHCs are geographically concentrated in the western world, as seen in Figure A1, with the majority of courses found in North America (54.03%) and Europe (34.27%). There was minimal course availability in Asia (4.44%), Oceania (4.03%) and South America (2.42%), and only 1 course was offered in Africa. Out of all the courses found, 191 (77.02%) were in countries identified as being in the top 10 contributors of global pollutants, while none were in countries ranked among the top 10 most vulnerable to the impacts of climate change.

### 3.2. Course Types and Modes of Delivery

Course types vary between universities and are accessible to different audiences, as indicated in Table A2.

#### 3.2.1. Undergraduate and Graduate Courses

Standalone courses represent the largest category of PHCs identified (98, 39.52%). The majority of these courses are offered at the graduate level only (52, 53.06%), with fewer available to undergraduate students (23, 23.47%) or to both groups (23, 23.47%). Undergraduate and graduate courses require formal admission and registration within a university and are part of a standard post-secondary education degree, such as a bachelor’s (undergraduate), master’s (graduate) or doctoral (graduate) degree. Some graduate courses have prerequisites for registration, such as being enrolled into a Master of Public Health, Global Health or Science. The availability of these courses is dependent on the university’s academic calendar, and some are not available every semester or school year.

#### 3.2.2. Undergraduate and Graduate Programs

Undergraduate programs refer to baccalaureate degrees that provide foundational education, while graduate programs include course-based or research-focused master’s and doctorate degrees, culminating in a final project or thesis. A total of 25 graduate programs (10.08%) offering comprehensive planetary health education and research were identified. There are two notable programs dedicated specifically to planetary health: the online Master’s Degree in Planetary Health offered jointly by the Universitat Oberta de Catalunya, Universitat Pompeu Fabra, and the Institute of Global Health, and the Master of Science in Planetary Health from the University of Edinburgh. Many master’s programs are concentrated in the United Kingdom (UK) and often combine planetary health with other disciplines, such as the MSc in Climate Change and Planetary Health at the London School of Hygiene & Tropical Medicine, MSc in Global and Planetary Health at Durham University, and the MSc in Global Environmental Change and Planetary Health at Cranfield University. Several programs combine planetary health and One Health, which brings increased attention to animal health considerations, such as at the University of Liverpool and the James Lind Institute [28]. There are also options to complete a minor or concentration in planetary health, which allow students to take specialized courses alongside their primary degree and earn additional certification. For example, Harvard T.H. Chan School of Public Health provides a Scholarly Concentration in Climate Change and Planetary Health to the Masters in Public Health (MPH), Masters of Science (MS), Doctor of Philosophy (PhD) or Doctor of Public Health (DrPH) students. At the doctoral level, four PhD programs were found, including the Doctor Programme in Planetary Health at the University of Lisbon and a PhD in Environmental and Planetary Health Sciences at the City University of New York. Undergraduate programs are limited, with only 5 programs (2.02%) identified, including the Global Challenges BASc with a focus on planetary health at Brunel University of London and two minor programs at the Dominican University of California and Pompeu Fabra University.

#### 3.2.3. Postdoctoral Fellowship

A single post-doctoral program was identified. The Planetary Health Postdoctoral Fellowship at Stanford University is designed for researchers who have completed their PhD and are looking to pursue independent, interdisciplinary research about planetary health. Researchers from diverse backgrounds are eligible to apply and are provided with funding to investigate the intersection between human health and the environment in areas such as mental health & resilience, air quality and health, and ecology, agriculture and infectious disease [29].

#### 3.2.4. Medical School Courses and Programs

PHCs in medical school programs are typically accessed as electives, which vary in duration and are available to students during specific elective periods within their academic calendar. Of all courses, thirty-nine (15.73%) medical school courses and programs were identified. In Canadian and U.S. medical schools, these typically occur during the pre-clinical years or the final year of study. Of these courses, eight specifically included “planetary health” in the course title, while others included it in the course description or syllabus. The Icahn School of Medicine at Mount Sinai and the New York Medical College courses focus on pediatric environmental health, which includes content related to how impacts of climate change can affect children’s health and development.

In addition to elective courses, six institutions offer scholarly concentrations, distinctions or pathway programs, which function similarly to minor programs by allowing students to take part in specialized, inquiry-based learning throughout the duration of their medical education. These are offered by the University of Ottawa, Rutgers University, Case Western Reserve University, Brown University and the University of North Carolina (UNC). Of these, the programs at Brown and UNC specifically feature “planetary health” in their titles.

#### 3.2.5. Open Access Courses and Certificate Courses

Thirty (12.10%) open access courses and 25 (10.08%) certificate courses were identified. Open access courses are free, asynchronous modules that can be accessed by anyone and do not require registration with a university. These courses are often offered in the form of Massive Open Online Courses (MOOCs), allowing learners to enroll freely and complete modules at their own pace. Certificate courses follow similar guidelines and generally do not require university enrollment. However, some are more structured, are offered in person, require certification fees, and are delivered synchronously. These courses are often hosted on online educational platforms, such as Coursera, edX and Future Learn. Notable examples include a series of open-access courses offered by CASCADES, a federally funded pan-Canadian capacity-building initiative for healthcare sustainability [23], as well as PHCs covering various areas of care provided by TelessaúdeRS-UFRGS, an innovative organization dedicated to improving healthcare in Brazil [30].

#### 3.2.6. Professional Development Courses

Professional development courses are designed to help current health professionals expand their knowledge of planetary health, specifically as it relates to their clinical roles and contexts. Twenty-five (10.08%) courses were identified, most similar in format to certificate courses, but some exclusive to healthcare workers in a particular health authority or association. The Centre for Sustainable Healthcare in England provides specialized training for various healthcare fields, including anesthetics, pediatrics, dentistry and respiratory care. Harvard offers a course for physicians, physician assistants and nurse practitioners to learn about how climate change impacts the healthcare delivery system—specifically diagnosis, counseling and treatment plans—while the Royal College of General Practitioners in the United Kingdom provides PHCs for their members. There are also courses for nurses, such as Yale University’s Planetary Health for Nurses course and a series of certificate courses from the Alliance of Nurses for Health Environment, which provide actionable steps to address the impacts of climate change on healthcare systems.

### 3.3. Course Duration

The duration of courses varies significantly based on the type of program. As displayed in Table A3, the majority of courses are offered over one semester (33.47%), which is the length of most undergraduate and graduate courses (15–17 weeks or 4 months). Forty-nine courses last 2 years or more (19.76%), and include undergraduate, doctoral and medical school programs, while courses lasting 1–2 years, primarily master’s programs, accounted for 8.06% of the offerings. Several courses found could be completed in less than one week (21.37%), and include short courses such as certificates, open access courses and professional development courses. Some courses allow students to enroll at any time and complete modules at their own pace, while others have structured timelines for course completion and therefore have firm deadlines for registration.

### 3.4. Tuition Costs

The courses exhibit a wide range of tuition fees, as depicted in Table A4, and vary based on whether a student was domestic or international, part-time or full-time, and the number of years the student took to complete the program.

Forty-nine free courses (19.76%) were identified, most of them open-access and certificate courses. The majority of undergraduate and graduate courses cost between $10,000–$25,000 USD, while undergraduate and graduate programs are usually over $50,000 USD. Twenty-one courses (8.47%) adjust tuition fees based on the student’s profession or country of origin which increased course accessibility for individuals from low- and middle-income countries (LMIC), which are classified by the World Bank Group based on the country’s gross national income [31]. The Virtual Global Health elective from the Child Family Health International NGO, for instance, costs $250 USD for students from LMICs versus $495 USD for students from high-income countries (HIC), and full scholarships are available for LMIC students. This scaling tuition fee structure is present in several high-level programs, such as the MPH in One Health and Planetary Health from James Lind Institute, which is $4093 USD (LMIC) versus $6253 USD (HIC).

### 3.5. Language Availability

The identified courses are primarily delivered in English (87.90%). A few courses are offered in German (4.03%), Spanish (2.42%), French (1.61%), Portuguese (0.81%), Catalan (0.81%), Dutch (0.81%), Turkish (0.81%), Italian (0.40%) and Swedish (0.40%). One exception is the Planetary Health for Nurses course at Yale University, which is delivered online through Coursera and provides instruction in a multitude of languages, including English, Dutch, Arabic, Bahasa Indonesia, French, Spanish, and Thai through auto-translation.

### 3.6. Surgical Content

The environmental scan identified four (2%) courses that explicitly addressed surgical content in the context of planetary health and sustainability. The course with the most surgery-based information is CASCADES’ Applied Fundamentals of Sustainable Health Systems: Sustainable Perioperative Care course, which aims to create advocates for more environmentally sustainable perioperative care. This course educates learners on the substantial impact of surgery on climate change, includes lectures from perioperative care providers leading sustainability efforts around the world and supports participants in developing quality improvement projects they can implement in their healthcare settings. The Centre for Sustainable Healthcare in England offers two relevant online certificate courses: Sustainable Anaesthetics, which explores topics such as anaesthetic gases, reducing the impact of general anaesthesia, and sustainable practices in operating departments, and Sustainable Child Health, which includes modules on sustainable surgery and operational aspects such as procurement, waste, and the use of single-use versus reusable devices. Emory University provides an online course for medical students, Climate Crisis and Clinical Medicine: Virtual Elective for Medical Students, which addresses strategies to mitigate the impact of operating rooms on the environment.

## 4. Discussion

### 4.1. Disparities in Geographical Distribution

Our environmental scan identified 248 planetary health educational opportunities spanning 32 countries. There was a high concentration of courses in the high-income countries that are major contributors to climate change, but we found very few in Africa, Asia and South America, almost none in LMICs, and none in countries most vulnerable to climate change. These countries are characterized by low climate readiness, which is the limited ability to prepare for and manage climate threats, and high fragility, which refers to the significant risk of disruption to government operations and public service systems [27]. While 7 out of the 10 countries most vulnerable to climate changes are situated on the African continent, our review found only one planetary health course in this region, offered at the graduate level in Rwanda: the MSc in Global Health Delivery at the University of Global Health Equity [27]. The inaccessibility of PHCs in areas most prone to consequences of climate change contributes to lower levels of environmental health literacy, in turn directly affecting the communities’ understanding of how environmental factors influence health [21,22,32]. The lack of courses tailored to the local environment, economy and culture can further hinder the implementation of effective sustainability measures, especially within healthcare systems. This finding is another illustration of the inequity in global health education, where countries in the Global North control the creation and distribution of knowledge, while countries in the Global South are not included in the development of these educational programs and have limited access to them [20].

Climate change disproportionately impacts countries with fewer socio-economic resources, which may be due to their reliance on climate-sensitive sectors such as agriculture and fishing, as well as by a lack of institutional, financial, and human resources to navigate climate change-related disasters [33]. Consequences of global warming include floods, droughts, heat waves, and melting glaciers [34], and a spike in the incidence of diseases transmitted by vectors, water, and food [35]. As a result, climate change adaptation should be a critical priority in regions where addressing climate issues is vital for community well-being and survival [36]. Consequently, and most importantly in LMICs, the development of context-relevant educational resources that focus on building climate resilience, strengthening local health systems and implementing evidence-based adaptation strategies is key. On the policy level, while LMICs produce 8.4 times fewer healthcare emissions than HICs, they have nonetheless demonstrated a clear commitment to low GHG emissions goals, as evidenced by their participation in the COP26 Health Programme [3,4]. However, these commitments have not yet translated into accessibility of planetary health education, as demonstrated by our scan finding only 13 courses in LMICs. A few of these courses specifically address planetary health in the context the students are in. The Sunway University course on Planetary Health and Sustainability in the Tropics and the Planetary Health and Global Climate Change in Costa Rica, a field-based program, demonstrate the value of regionally contextualized education. In contrast, the majority of the PHCs in North America and Europe provide very general content, not necessarily translatable to those fragile settings. Scaling these models in countries most vulnerable to climate change could allow for the development of effective, context-based solutions that empower communities to better address climate change. Addressing these disparities is crucial to building a planetary health workforce and combating the detrimental impacts of climate change.

### 4.2. Integration of Planetary Health Education into Health Professional Training

Our environmental scan found that the majority of PHCs are offered at the graduate level, with few available to undergraduate students or as a formal component of medical school curricula. This trend demonstrates how planetary health is often seen as an advanced specialization or optional course, rather than as a foundational competency, leading to a lack of investment in early planetary health education. Although full master’s programs are valuable for specialized, comprehensive training, they are only accessible to a subset of learners with directed interests in planetary health who have already completed early education stages.

In 2022, most of the world’s medical schools (85%) did not include planetary health in their curricula [37] but there has since been a significant rise in medical schools’ commitment to advancing climate education, in tandem with growing calls from the academic community [38]. Of the 139 reports submitted to the 2025 Planetary Health Card initiative, 42 came from newly participating medical schools, demonstrating growing engagement in planetary health education from global institutions. For instance, in 2023, the University of British Columbia embedded planetary health content into two required courses on clinical education and medical practice [39], incorporating patient and caregiver perspectives to empower students to be advocates for sustainability [40]. Healthcare leaders have called for the integration of planetary health in the foundations of medical curricula, as seen in Simon Fraser University’s new medical school being built on planetary health principles [41] and efforts across Harvard University to introduce more climate education resources [42]. Though majority of medical schools integrating planetary health education are concentrated in North America, there is a growing global movement led by medical students for broader adoption of climate change and health curricula worldwide [43]. In Europe, the University of Glasgow led the establishment of the European Network in Climate and Health Education in 2024, which works with 42 medical schools to train students to mitigate the effects of climate change on health and use more sustainable practices [44]. A similar network has been established in South America by University of Concepción, called the Chilean Planetary Health Network, which works on research, student training and community engagement related to climate change and health [45]. Progress in climate education remains slower in the Global South, but health systems are starting to implement strategies to provide the critical training to current and future physicians [46]. In India, 68.9% of medical schools lack climate-related education in their program [47], despite the country’s status as one of the world’s largest polluters [26] and it’s frequent exposure to extreme environmental events, such as heatwaves and floods [48]. St. John’s Medical College in Bengaluru, India stands out with its mandatory “Citizen Doctor” course, which promotes environmental responsibility among medical students [49]. The United Nations is also working with national governments to support health systems in countries most vulnerable to climate change. With support from the World Health Organization and United Nations Development Programme, Bangladesh, Nepal and Laos developed climate change and health resources that have been disseminated nationwide to educate healthcare students and professionals [46].

Although the integration into core curricula is growing, it remains limited and difficult to assess, with some schools offering very minimal content such as a single lecture, a short online module or a paper recommendation referencing planetary health. The courses identified through the environmental scan suggest that content is still limited to electives or minor programs, making it more likely that only students with prior knowledge or a specific interest in the topic will engage with these learning opportunities. However, student-driven efforts, such as involvement in the Planetary Health Report Card initiative and reports from medical student groups, indicate a clear demand from students to see planetary health formally embedded throughout their training rather than as an optional area of study [50].

The integration of planetary health into early healthcare education allows health professionals to address the interconnected health of patients and the planet, and advocate for environmentally informed and environmentally preferable care [37,51,52]. Moreover, health professionals can educate the public and use their influence to establish policies aimed at protecting the environment, in addition to implementing measures in their professional, personal, and institutional practices to reduce GHG emissions [53]. Hence, educational institutions, program administrators, healthcare organizations and governments need to urgently collaborate to integrate planetary health content across all levels of health professional education. At a policy level, formally integrating sustainability as a dimension of quality of care can support and facilitate the transition from knowledge to action for healthcare providers, further accelerating progress towards low carbon, resilient and sustainable healthcare systems [54,55,56,57,58].

### 4.3. Accessibility Barriers

There are several barriers to accessing planetary health education beyond geographical distribution, including cost, language availability and course duration. The majority of courses are delivered exclusively in English, which excludes many learners, especially those in regions with lower English proficiency. This reinforces the need for context-based educational resources that are offered in local languages to ensure equitable access and effective knowledge transfer between regions.

Duration also plays a role in accessibility, as full-time, in-person, and long courses can be challenging for healthcare professionals to complete. One survey assessed the perspective of healthcare professionals from 12 institutions around the world on the impacts of climate change on human health. The authors found that the main barrier to the respondents’ engagement in climate change and health was time constraints, despite their high level of understanding of the importance of the issue and their willingness to advocate on the subject [53].

The cost of educational opportunities is also a major barrier for many prospective learners, particularly those from low-income backgrounds or resource-limited regions [59,60]. Among the courses we identified, undergraduate and graduate programs, which provide the most comprehensive training, were the most difficult to access, as they were associated with the highest financial commitments and the longest duration. While course fees were lower for part-time enrolment, the overall costs for participants were higher given the longer duration of studies. These fees also do not consider the opportunity costs associated with lost income due to full-time enrollment, which may be prohibitive depending on participants’ career stage and personal circumstances. A recent study advocated for free planetary health education, especially for healthcare professionals [61]. Although this may be difficult to implement for all courses, studies have highlighted the potential use of sliding scale tuition models to promote greater equity in access to higher education, by adjusting fees based on a student’s financial capacity [62]. Some courses had variable fee structures based on whether the participant’s country of origin is an HIC or LMIC, which allowed for more equitable access. This approach is particularly impactful in the context of planetary health and sustainable surgical education, where equitable access is essential for building global capacity to address climate change. Implementing a flexible and needs-based fee structure in PHCs could help mitigate financial barriers, ensuring that healthcare professionals, especially those in low- and middle-income countries, can access critical training needed to reduce the GHG emissions of healthcare systems and respond effectively to environmental challenges. Some courses also overlapped across categories. For example, a course could be both a certificate program and open access, offering learners the option to pay a fee to obtain official credentials of course completion.

Another opportunity could be to make PHCs more widely available online. Studies have shown that online courses for health professionals bring several benefits, such as greater flexibility and knowledge management, easier access and greater cost-effectiveness [63]. Examples of such opportunities include the open access courses delivered by TelessaudesRS-UFRGS [30] and the course series from CASCADES [64], which are accessible, free and provide a reliable foundation for creating additional educational resources [61]. This format democratizes educational resources, allowing learners to participate regardless of their geographical location, institutional affiliation, and financial limitations.

### 4.4. Gaps in Sustainable Surgery Education

Out of the 248 courses, only 4 courses mentioned surgery, revealing an important gap. The Sustainable Anaesthetics course from the Centre for Sustainable Healthcare (Oxford, England) had the most comprehensive surgical content, assessing all the different components of surgical care and its environmental. The remaining three courses were primarily focused on broader subjects and offered some context-based information about sustainability in surgery, such as anesthesia and medical waste. Many other courses addressed sustainable healthcare more broadly, covering topics such as facilities management, greenhouse gas accounting, and identifying carbon hotspots; however, none mentioned surgery in their course pages, syllabi, or learning objectives.

Despite the growing emphasis on planetary health, surgical practice remains largely absent from these discussions—and conversely, environmental sustainability has yet to be meaningfully integrated into surgical care. Operating rooms are among the most resource-intensive components of healthcare, contributing to energy consumption, anesthetic gas emission and waste production. Incorporating surgical content into planetary health education provides a way to develop strategies that clinicians can apply in their daily practice, enabling them to deliver high-quality patient care while minimizing environmental impact. These efforts also link to broader public health goals by emphasizing preventive measures that reduce the need for surgical care and improving population health.

To increase surgical capacity in LMICs, dozens of National Surgical Obstetrics and Anesthesia Plans (NSOAPs) are being written and implemented. In a recent Lancet Planetary Health commentary, Nazir et al. emphasized the near absence of environmental sustainability within published NSOAPs [65]. This is a cause for concern, since operating rooms contribute significantly to climate change [65,66], yet LMICs need to drastically increase their surgical capacity while also protecting their environment. A recent publication estimated that increasing the number of yearly operations by 143 million in LMICs in order to meet the Lancet Commission on Global Surgery targets would result in approximately 858 million to 116.4 billion kg of CO2e produced per year [65,67,68]. It is therefore critical to educate surgical professionals to support the implementation of sustainable practices such as renewable energy sources in operating rooms, sustainable anesthetics and waste reduction, in tandem with supportive policy and infrastructural changes [69,70]. A recent survey of 450 surgeons across 55 countries revealed that only 40% were aware of sustainable practices in the operating room. This finding underscores the widespread lack of awareness among surgeons, as well as the insufficient guidance and leadership provided by institutions, governments, policymakers, and health agencies, factors that collectively hinder progress toward establishing environmentally sustainable operating rooms [71,72]. Consequently, we strongly recommend that hospitals and educational institutions implement PHCs that address the impact of surgery on the environment. These courses could include hands-on, quality improvement projects and case-based learning, and consider strategies to promote sustainable surgical care using mitigation and adaptation practices.

### 4.5. Limitations

These findings indicate an urgent need to integrate planetary health education into all levels of health professional training, to prioritize course development in vulnerable regions, and to provide multilingual, open-access resources to ensure equitable access. To our knowledge, this is the first study to highlight the absence of surgical content within PHCs worldwide. The environmental scan was carried out using three different data collection methods, which had methodological limitations that may have affected the comprehensiveness and accuracy of our data collection. Our initial search strategy relied on Google searches with simple keywords, which may have excluded courses containing content related to planetary health that was embedded in curricula or not explicitly labeled with the keywords. As the searches were conducted primarily in English, courses offered exclusively in other languages may not have been captured, limiting representation from non-English speaking regions. Specifically in medical schools, planetary health content may sometimes be limited to a single lecture, seminar or reading assignment, making it difficult to identify through our search methodology. The Planetary Health Report Card [25] provided valuable information about curriculum integration in health professional schools, but collecting this information was challenging as our search focused on formal courses that could be verified through institution websites rather than individual lectures. Additionally, full course duration and tuition data were collected for medical school programs as access to the planetary health course requires enrollment in the entire program. However, this method may overestimate the duration and cost of the course when the planetary health content represents only a small portion of the entire degree. Moreover, for most courses, only summaries and learning objectives were available, which made it challenging to fully evaluate whether they included surgery-related content. There may be courses with content around surgery and planetary health that could be accessed through course enrollment and completion but were not identified through our methods. Although course professors and department heads were contacted through email to gain further insight into courses, it was difficult to attain all the course information. This study also focused on course availability rather than assessing the quality, outcomes or impact of these programs, which is essential to understand the effectiveness of the content and to inform future development of PHCs. Future research should employ more comprehensive search strategies, further investigate embedded content, and study the long-term effects of planetary health education on learner outcomes and healthcare practices.

### 4.6. Future Directions

Further development of accessible courses in regions vulnerable to climate change that provide context-based resources for incorporating sustainable measures into healthcare practices is urgently required. Increased dissemination of existing high-quality courses, in the form of free, open-access, online courses available to learners globally, should be supported. Education teams of instructional designers, subject experts and content writers can use existing planetary health syllabi as the basis for developing new courses [73].

Integrating planetary health content into health professional education is essential for building sustainable healthcare systems that can address the impacts of climate change. Early, accessible and contextually relevant education is crucial for advancing sustainable healthcare systems globally. Including surgical content is also extremely important, as the operating room is a major contributor to the healthcare system’s overall carbon footprint. Hospitals, universities, and accreditation bodies should prioritize the inclusion of planetary health in core medical education, ensuring that all health professionals are equipped with the knowledge and skills to reduce the environmental footprint of healthcare. The Planetary Health Report Card can be used to evaluate current planetary health content available in healthcare programs [18], and course improvement or development can be performed using the framework from the 2030 Sustainable Development Goals from the UN, which outlines how to integrate sustainability into health professions curricula [74].

Government involvement is also essential for achieving sustainability in healthcare, as educational goals and major curriculum changes require coordination across different levels of public health agencies and health authorities. An example of this is the National Health Service (NHS) in the UK, which is mandated by the Health and Care Act 2022 to reach net zero GHG emissions [75]. The UK government has provided guidelines and educational resources for health professions to act on the climate crisis in their professional practice [76]. Initiatives such as the NHS’s sustainability training sessions and the planetary and global health courses delivered by the Royal College of General Practitioners demonstrate how government and healthcare institutions can work together to efficiently integrate sustainability into healthcare systems. Finally, educated providers must be enabled to enact the necessary transformations, which will require strong governmental support through policies and monitoring [4,77].

## 5. Conclusions

Planetary health education is expanding but remains largely concentrated in high-income countries and is predominantly offered in English, at the graduate level. Accessibility continues to be limited by barriers such as cost, time, and language, highlighting the importance of developing asynchronous, low-cost, and multilingual formats to broaden reach. The limited availability of surgical and perioperative content indicates an opportunity to integrate context-based modules focused on quality improvement to enhance the relevance and scalability of planetary health education worldwide.

## Data Availability

All data are available in the manuscript, appendix and Supplementary Materials. Further inquiries can be directed to the corresponding author.

## Supplementary Materials

The following supporting information can be downloaded at: https://www.mdpi.com/article/10.3390/ijerph22101545/s1, File S1: Planetary Health Course Data Sheet.

## Abbreviations

The following abbreviations are used in this manuscript:

PHC	Planetary Health Course
OR	Operating Room
LMIC	Low- and middle-income country
HIC	High-income country
GHG	Greenhouse gas

## Appendix A

**Table A1:** Geographical Distribution of Courses.

Regions	# of Courses	%
North America	134	54.03
Europe	85	34.27
Asia	11	4.44
Oceania	10	4.03
South America	6	2.42
Africa	1	0.40
Global	1	0.40

**Table A2:** Type of Course.

Course Type	# of Courses	%
Undergraduate Course	23	9.27
Graduate Course	52	20.97
Undergraduate and Graduate Course	23	9.27
Undergraduate Program	5	2.02
Graduate Program	25	10.08
Medical School	39	15.73
Professional Development Course	25	10.08
Certificate	25	10.08
Open Access Course	30	12.10
Postdoctoral Fellowship	1	0.40

**Table A3:** Course Duration.

Duration	# of Courses	%
Less than 1 week	53	21.37
Less than 1 month	12	4.84
1 month	6	2.42
2–4 months	18	7.26
1 Semester	83	33.47
5 months–1 year	2	0.81
1–2 years	20	8.06
2+ years	49	19.76
Missing	5	2.02

**Table A4:** Tuition Fees.

Cost	# of Courses	%
Free	49	19.76
Less than $1000 USD	12	4.84
$1000–$5000 USD	21	8.47
$5000–$10,000 USD	25	10.08
$10,000–$25,000 USD	53	21.37
$25,000–$50,000 USD	17	6.85
$50,000+ USD	44	17.74
Variable based on country or profession	21	8.47
Missing	6	2.42

**Table A5:** Language Availability.

Available Languages	# of Courses	%
Only English	218	87.90
German	10	4.03
Spanish	6	2.42
French	4	1.61
Portuguese	2	0.81
Catalan	2	0.81
Dutch	2	0.81
Turkish	2	0.81
Italian	1	0.40
Swedish	1	0.40

## Appendix B

### Appendix B.2. Inclusion and Exclusion Criteria

Courses were considered for inclusion if they featured a structured curriclum with clearly defined learning objecives, organized content and/or an evaluation process or credits/certifcate upon completion. Eligible courses had to primairly focus on content related to planetary health, climate change and health, environment and health, or sustainable health practices. Delivery of courses could be in any format, and could be delieverd by educational instutitions, MOOCs or global organizations. Exclusion criteria ruled out informal workshops, seminars without assessment or certification, lectures integrated within a course unrelated to planetary health and sustainabitliy, and programs lacking explicit reference to climate change or environment and health in their objectives, description or syllabi.

### Appendix B.3. Google Search Strategy

A targeted Google search approach was used to identify PHCs, simulating a search that students and health professionals would use when seking specfici educational opportunities online. The search was conducted in December 2022 using the term “planetary health courses”, and only the first ten pages of results were reviewed to ensure relevance to the topic. This method allowed for courses across multiple discipliens and geopgrahic regions to be captured, including smaller programs or certificates that do not appear in standard academic databases.

### Appendix B.4. Author Data Review

The courses were collected and verified by multiple authors. Intial collection, screening and data accuracy review of courses from the curriculum scan of top 10 universities in each global region, the Google search and Asaduzzaman et al. (2022) was conducted by R.V., P.Z.M. and A.Z.M. [20]. Courses obtained from the Planetary Health Alliance Global Inventory of PHCs and the 2025 Planetary Health Report Cards were collected, screened and reviewed by R.V., L.Q.C. and R.J., R.V. conducted a final review of all courses prior to submission in June 2025 to ensure consistency in reporting data and the correct application of the inclusion and exclusion criteria.
