# Supplementary material for: Surgical Education Within Planetary Health Curricula: A Global Environmental Scan (2022–2025)
Source: Int J Environ Res Public Health. 2025 Oct 10;22(10):1545. doi: 10.3390/ijerph22101545 (PMC12564437; doi:10.3390/ijerph22101545)
Supplement: Supplementary file 1 [file ijerph-22-01545-s001.zip › Supplementary - Planetary Health Course Data Sheet.pdf]

Region	Country	Institution Name	Course name	Format	Course Type	Available Languages	Length	Costs ($USD)	Is country top 10 most polluting?	Is country top 10 most vulnerable to climate change?	Relationship between planetary health and surgery addressed?	If yes, what:	Link
Africa	Rwanda	University of Global Health Equity	MSc Global Health Delivery - One Health track	In Person	Graduate Program	English	*Missing	*Missing	No	No	No		https://ughe.org/master-of-science-in-global-health-delivery-mghd/
Asia	China	Duke Kunshan University	Planetary Health and Environmental Epidemiology	In Person	Graduate Course	English	1 Semester	$63,450.00	Yes	No	No		https://env.dukekunshan.edu.cn/videos-library/planetary-health-and-environmental-epidemiology-john-ji/ (Inactive)
Asia	China	Duke Kunshan University	Global Environmental Health Problems: Principles and Case Studies	Online	Graduate Course	English	1 Semester	$63,450.00	Yes	No	No		https://nicholas.bulletins.duke.edu/courses/0245661
Asia	Japan	Institute of Science Tokyo	Master of Public Health in Global Health	In Person	Graduate Program	English	2 years	$8,827.02	Yes	No	No		https://www.tmd.ac.jp/cmn/mphgh/
Asia	Japan	Hiroshima University	Development and Culture: Introduction to Planetary Health	In Person	Graduate Course	English	1 Semester	$1,860.85	Yes	No	No		https://momiji.hiroshima-u.ac.jp/syllabusHtml_en/2025_E50106_WMG02901_en.html
Asia	Japan	Nagasaki University	Doctor of Public Health	In Person	Graduate Program	English	3 years	$3,397.91	Yes	No	No		https://www.planetaryhealth.nagasaki-u.ac.jp/en/drph-program/
Asia	Malaysia	Sunway University	Planetary Health and Sustainability in the Tropics	In Person	Undergraduate Course	English	1 month	$2,850.00	No	No	No		https://sunwayuniversity.edu.my/school-of-medical-life-sciences/courses/planetary-health-and-sustainability-tropics
Asia	Phillipines	University of the Philippines Los Baños	Ecology and Planetary Health	Online	Undergraduate Course	English	1 Semester	$752.43	No	No	No		https://sites.google.com/view/bio191bsph/home
Asia	Thailand	Thammasat University	Globilization Flows	In Person	Undergraduate Course	English	1 semester	$2,700.00	No	No	No		https://sgs.tu.ac.th/wp-content/uploads/2023/10/GSSE-curriculum-2023.pdf
Europe	Denmark	University of Copenhagen	Drivers of Change in Human Health: Coping with population and environmental dynamics	In Person	Graduate Course	English	1 semester	$6,150.71	No	No	No		https://kurser.ku.dk/course/SGLK15002U/2023-2024
Europe	Denmark	University of Copenhagen	Master of Disaster Management program	In Person	Graduate Program	English	1 year	$23,994.30	No	No	No		https://www.mdma.ku.dk/programme-structure/
Europe	England	Brunel University London	Global Challenges (Planetary Health) BASc	In Person	Undergraduate Program	English	3-4 years	$83,148.99	Yes	No	No		https://www.brunel.ac.uk/study/courses/global-challenges-basc
Europe	England	Durham University	MSc Global and Planetary Health	In Person	Graduate Program	English	1-2 years	$37,878.03	Yes	No	No		https://www.durham.ac.uk/study/courses/global-and-planetary-health-l6ka07/
Europe	England	London School of Hygiene and Tropical Medicine	MSc Climate Change & Planetary Health	In Person or Online	Graduate Program	English	1 year	$19,902.88	Yes	No	No		https://www.lshtm.ac.uk/study/courses/masters-degrees/climate-change-planetary-health
Europe	England	Imperial College London	Climate change and health	Online	Open Access Course	English	56 hours	Free	Yes	No	No		https://www.coursera.org/learn/climate-change-health
Europe	England	ET Health via Future Learn	Impact of Climate Change on Public Health	Online	Open Access Course	English	4 weeks	Free	Yes	No	No		https://www.futurelearn.com/courses/climate-change-public-health
Europe	England	University of Exeter	MSc Environment and Human Health	In Person	Graduate Program	English	1-2 years	$37,998.06	Yes	No	No		https://www.exeter.ac.uk/study/postgraduate/courses/medicine/environment-health-msc/
Europe	England	Centre for Sustainable Healthcare	Carbon Footprint for Healthcare	Online	Professional Development Course	English	10 hours	$203.82-$445.85, Variable, based on salary	Yes	No	No		https://sustainablehealthcare.org.uk/courses/carbon-footprinting-healthcare
Europe	England	Centre for Sustainable Healthcare	Green Spaces and Health	Online	Professional Development Course	English	10 hours	$127.38-$318.46, Variable, based on salary	Yes	No	No		https://sustainablehealthcare.org.uk/courses/green-space-and-health
Europe	England	Centre for Sustainable Healthcare	Introduction to Sustainable Healthcare	Online	Professional Development Course	English	10 hours	$127.38-$318.46, Variable, based on salary	Yes	No	No		https://www3.copernicus.co.uk/SHLCLassic/Default
Europe	England	Centre for Sustainable Healthcare	Public Health Leadership for Sustainability	Online	Professional Development Course	English	10 hours	$127.38-$318.46, Variable, based on salary	Yes	No	No		https://sustainablehealthcare.org.uk/courses/public-health-leadership-sustainability
Europe	England	Centre for Sustainable Healthcare	Sustainability Quality Improvement	Online	Professional Development Course	English	10 hours	$203.82-$445.85, Variable, based on salary	Yes	No	No		https://sustainablehealthcare.org.uk/courses/sustainability-quality-improvement
Europe	England	Centre for Sustainable Healthcare	Sustainable Anaesthetics	Online	Professional Development Course	English	10 hours	$127.38-$318.46, Variable, based on salary	Yes	No	Yes	- Anaesthetics: gases, desflurane, nitrous oxide, reducing the impact of general anaesthesia, total intravenous anaesthesia, regional anesthesia<br>-The operating department: green hospitals, anaesthetic rooms and theatres, procurement, waste, single vs reusable devices, PPE and pandemic, travel, telemedicine and patient self-monitoring apps	https://sustainablehealthcare.org.uk/courses/sustainable-anaesthetics
Europe	England	Centre for Sustainable Healthcare	Sustainable Child Health	Online	Professional Development Course	English	10 hours	$127.38-$318.46, Variable, based on salary	Yes	No	Yes	- Sustainable Healthcare in Practice: Sustainable surgery<br>-Operational Aspects: procurement, waste, single use vs reusable devices, PPE and pandemic, travel,, energy	https://sustainablehealthcare.org.uk/courses/sustainable-child-health
Europe	England	Centre for Sustainable Healthcare	Sustainable Dentistry	Online	Professional Development Course	English	10 hours	$127.38-$318.46, Variable, based on salary	Yes	No	No		https://sustainablehealthcare.org.uk/courses/sustainable-dentistry

Region	Country	Institution Name	Course name	Format	Course Type	Available Languages	Length	Costs ($USD)	Is country top 10 most polluting?	Is country top 10 most vulnerable to climate change?	Relationship between planetary health and surgery addressed?	If yes, what:	Link
Europe	England	Centre for Sustainable Healthcare	Sustainable Kidney Care	Online	Professional Development Course	English	10 hours	$127.38-$318.46, Variable, based on salary	Yes	No	No		https://sustainablehealthcare.org.uk/courses/sustainable-kidney-care
Europe	England	Centre for Sustainable Healthcare	Sustainable Mental Healthcare	Online	Professional Development Course	English	10 hours	$127.38-$318.46, Variable, based on salary	Yes	No	No		https://sustainablehealthcare.org.uk/courses/sustainable-mental-healthcare
Europe	England	Centre for Sustainable Healthcare	Sustainable Primary Care	Online	Professional Development Course	English	10 hours	$127.38-$318.46, Variable, based on salary	Yes	No	No		https://sustainablehealthcare.org.uk/courses/sustainable-primary-care
Europe	England	Centre for Sustainable Healthcare	Sustainable Procurement	Online	Professional Development Course	English	10 hours	$127.38-$318.46, Variable, based on salary	Yes	No	No		https://sustainablehealthcare.org.uk/courses/sustainable-procurement
Europe	England	Centre for Sustainable Healthcare	Sustainable Respiratory Care	Online	Professional Development Course	English	10 hours	$127.38-$318.46, Variable, based on salary	Yes	No	No		https://sustainablehealthcare.org.uk/courses/sustainable-respiratory-care
Europe	England	Centre for Sustainable Healthcare	Teaching Sustainability in Quality Improvement	Online	Professional Development Course	English	6.5 hours	$127.38	Yes	No	No		https://sustainablehealthcare.org.uk/courses/teaching-sustainability-quality-improvement
Europe	England	Durham University	Planetary Health in Social Context	In Person	Graduate Course	English	1 Semester	18 939.015	Yes	No	No		https://rl.talis.com/3/durham/lists/14E4AFF0-8C8F-5C9D-BC1F-284AC1D70D37.html?lang=en-GB
Europe	England	University of Cambridge	MPhil Population Health Sciences - Global Health Specialisation	In Person	Graduate Program	English	10 months full time, 22 months part time	$74,507.65	Yes	No	No		https://www.postgraduate.study.cam.ac.uk/courses/directory/cvphmpphs
Europe	England	University of Exeter	Master of Public Health	In Person	Graduate Program	English	1-2 years	$37,980.23	Yes	No	No		https://www.exeter.ac.uk/study/postgraduate/courses/medicine/mph/
Europe	England	London School of Hygiene and Tropical Medicine	Agriculture, Nutrition and Health	Online	Certificate	English	*Missing	$39.83	Yes	No	No		https://epay.lshtm.ac.uk/product-catalogue/open-study/certification/open-study-certification-agriculture-nutrition-and-health
Europe	England	Centre for Sustainable Healthcare	Sustainable Healthcare (cross speciality)	Online	Certificate	English	13 hours	$127.38-$318.46, Variable, based on salary	Yes	No	No		https://www3.copernicus.co.uk/SHL/contentviewer/20478/1/1/308/0
Europe	England	Cranfield University	Global Environmental Change and Planetary Health MSc	In Person	Graduate Program	English	1 year full time, 2-3 years part time	$15,795.76	Yes	No	No		https://www.cranfield.ac.uk/courses/taught/global-environmental-change-and-planetary-health#Overview
Europe	England	University of Liverpool	Planetary and One Health (MSc)	In Person	Graduate Program	English	1 year	$37,584.52	Yes	No	No		https://www.liverpool.ac.uk/courses/planetary-and-one-health-msc
Europe	England	Royal College of General Practioners	Introduction to sustainable healthcare	Online	Professional Development Course	English	0.5 hours	Free for RCGP members	Yes	No	No		https://elearning.rcgp.org.uk/course/view.php?id=650
Europe	England	Royal College of General Practioners	High quality & low carbon respiratory care	Online	Professional Development Course	English	0.5 hours	Free for RCGP members	Yes	No	No		https://elearning.rcgp.org.uk/course/view.php?id=650
Europe	Finland	University of Jyvaskyla	Planetary well-being studies	Online	Open Access Course	English and Finnish	*Missing	Free	No	No	No		https://www.jyu.fi/fi/avoin-yliopisto/opinnot/planetaarinen-hyvinvointi-opinnot-avoimessa-yliopistossa-maksuton
Europe	Finland	iaconia University of Applied Sciences, VID Specialized University (Norway), Carinthia University of Applied Sciences (Austria), Ede Christian University of Applied Sciences (Netherlands), Technical University of Applied Sciences Würzburg-Schweinfurt (Germany), University College Cork (Ireland) and Palawan State University (Philippines)	Planetary Health Blended Intensive Programme	Hybrid	Certificate	English	1 week	Free for students at partner universities	No	No	No		https://www.diak.fi/en/studying/international/intensive-programmes/blended-intensive-programmes-at-diak/planetary-health/
Europe	France	Conservatoire national des Arts et Metiers	Module de Sante Planetaire	Online	Graduate Course	English and French	6 hours	*Missing	No	No	No		https://formation.cnam-hauts-de-france.fr/formations/nue.php?id=5893
Europe	France	France Université Numérique	Medicine and environmental health	Online	Open Access Course	French	5 weeks	Free	No	No	No		https://www.fun-mooc.fr/fr/cours/medecine-et-sante-environnementale/
Europe	Germany	CIH LMU Munich and Planetary Health Academy (KLUG)	Climate change and planetary health initiating and leading transformative change	Online	Certificate	German	90 hours	$642.48	Yes	No	No		https://www.klimawandel-gesundheit.de/climate-change-and-planetary-health-initiating-and-leading-transformative-change/
Europe	Germany	Virtual University of Bavaira	Planetary Health Course	Online	Undergraduate Course	German	20 hours	Free	Yes	No	No		https://www.uni-augsburg.de/en/fakultaet/med/profs/klimawandel-gesundheit/forschung/planetary-health/
Europe	Germany	Universitat Bayreuth	Planetary Health, Public Health & Economics	In Person	Undergraduate Course	English	2 weeks	$1,204.44	Yes	No	No		https://www.summerschool.uni-bayreuth.de/en/courses/Public_Health/index.html
Europe	Germany	University of Augsburg, University of Bayreuth, University of Munich (LMU), University of Regensburg, University of Würzburg.	Planetary Health Gesunde Erde, gesunde Menschen	Online	Open Access Course	German	20 hours	Free	Yes	No	No		https://open.vhb.org/blocks/occoursemetaselect/detailpage.php?id=295#
Europe	Germany	University of Wurzburg, University of Bayreuth University of Eldoret, Catholic University of Health and Allied Sciences in Mwanza	Summer School Planetary Health	In Person	Certificate	English	10 days	Free	Yes	No	No		https://www.med.uni-wuerzburg.de/en/planetary-health/summer-school-planetary-health/
Europe	Germany	University of Munich (LMU),	Planetary Health Gesunde Erde, gesunde Menschen?	Online	Open Access Course	German	20 hours	Free	Yes	No	No		https://open.vhb.org/blocks/occoursemetaselect/detailpage.php?id=295

Region	Country	Institution Name	Course name	Format	Course Type	Available Languages	Length	Costs ($USD)	Is country top 10 most polluting?	Is country top 10 most vulnerable to climate change?	Relationship between planetary health and surgery addressed?	If yes, what:	Link
Europe	Germany	Ludwig Maximilian University	Climate Change and Health	Online	Open Access Course	English	5 days	HIC: $225.84 LMIC: $112.94	Yes	No	No		https://www.cih.lmu.de/education/short-term-courses/advanced-modules-in-international-health/climate-change-and-health
Europe	Germany	Universitat Bayreuth	Planetary Health	In Person	Undergraduate and Graduate Course	German	1 semester	$184.18	Yes	No	No		https://my.uni-bayreuth.de/cmlife/s/courses/Ly91YnRAY21jby9hcGkvY291cnNlcy8zNTQzODQ%3D/description
Europe	Germany	Universitat Bayreuth	Action Research for Planetary Health	In Person	Undergraduate and Graduate Course	English	1 semester	$184.18	Yes	No	No		https://my.uni-bayreuth.de/cmlife/s/courses/Ly91YnRAY21jby9hcGkvY291cnNlcy8zNTU4NTQ%3D/description
Europe	Ireland	Maynooth University	Master's in Environmental Psychology	In Person	Graduate Program	English	1 year	$19,182.05	No	No	No		https://www.maynoothuniversity.ie/news-events/new-msc-environmental-psychology-open-applications (Inactive)
Europe	Ireland	The Royal College of Surgeons in Ireland	Sustainable Healthcare	Online	Open Access Course	English	5 weeks	Free	No	No	No		https://www.rcsi.com/online/find-a-course/cpd/s/u/sustainable-healthcare
Europe	Italy	University of Turin	Specializing Master in Sustainability in Health	Online	Graduate Program	English	12 months	$2,727.00	No	No	No		https://www.sustainabilityinhealth.unito.it/do/home.pl/View?doc=/About/Program_Overview.html#:~:text=The%20International%20Master's%20Program%20in,environmental%20sustainability%20and%20human%20health. (Inactive)
Europe	Italy	University of Pavia	ADE Planetary Health: le sfide attuali e future della medicina pubblica	In Person	Undergraduate Course	Italian	8 hours	*Missing	No	No	No		https://www.ghislieri.it/project/planetary-health-le-sfide-attuali-e-future-della-medicina-pubblica/
Europe	Netherlands	EWUU Alliance	Planetary Health and Climate Resilient Health Systems	Hybrid	Graduate Course	English	6 weeks	*Missing	No	No	No		https://ewuu.nl/en/education/challenges/planetary-health-and-climate-resilient-health-systems-challenge/
Europe	Netherlands	Radboud University	Planetary Health Keuzevak	In Person	Undergraduate Course	Dutch	*Missing	*Missing	No	No	No		https://www.radboudumc.nl/over-het-radboudumc/strategie/themas/duurzaamheid/onze-pijlers/duurzaam-onderwijs-en-onderzoek/duurzaam-onderwijs-onderzoek/planetary-health
Europe	Netherlands	University of Medical Center Utrecht	Global and Planetary Health	Online	Medical School	English and Dutch	4 years	$90,708.12	No	No	No		Syllabus link https://www.dropbox.com/s/bv479zcj2h13cc1/GHEHCC%20syllabus%20BB%20v4b.pdf?e=1&dl=0
Europe	Netherlands	University of Twente	Geo-Health	Online	Certificate	English	10 weeks	$1,599.86	No	No	No		https://www.itc.nl/education/study-finder/geo-health-5ec/
Europe	Netherlands	University of Medical Center Utrecht	Online Course: Planetary Health	Hybrid	Open Access Course	English	10 hours	Free	No	No	No		https://www.globalhealth.eu/planetary-health/
Europe	Netherlands	University of Groningen	BSc Global Responsibility and Leadership, Major in Psychology and Global Health	In Person	Undergraduate Program	English	3 years	$67,165.71	No	No	No		https://ocasys.rug.nl/current/catalog/programme/59327
Europe	Netherlands	University of Groningen, University of Antioquia	Planetary Health - Epidemiology meets technology	Hybrid	Certificate	English	2 weeks	HIC: $976 LMIC: $759	No	No	No		https://www.rug.nl/education/summer-winter-schools/planetary-health/?lang=en
Europe	Netherlands	Utrecht University	Planetary Health: Towards Just Futures	In Person	Graduate Course	English	5 days	HIC: $954.31 LMIC: $615.51	No	No	No		https://utrechtsummerschool.nl/courses/life-sciences/planetary-health-towards-just-futures
Europe	Netherlands	University of Groningen	Collaborating in Planetary Health	Online	Undergraduate and Graduate Course	English	21 days	Free for students at ENLIGHT Alliance unvierisities	No	No	No		https://enlight-eu.org/students/courses/1177-planetary-health
Europe	Netherlands	University of Twente	Geospatial for Planetary Health	Online	Open Access Course	English	5 hours	Free	No	No	No		https://www.geoversity.io/courses/geospatial-planetary-health/
Europe	Portugal	University of Lisbon	Planetary Health Studies - Doctoral Program	In Person	Graduate Program	English	4 years	$12,419.72	No	No	No		https://www.ulisboa.pt/en/planetary-health-studies
Europe	Scotland	University of Edinburgh	MSc in Planetary Health or PG Certificate	In Person or Online	Graduate Program	English	1 year	MSc: $35,989.14 PG Cert:$13,505.89	Yes	No	No		https://efi.ed.ac.uk/programmes/planetary-health/?gad_source=1&gad_campaignid=21861637163&gbraid=0AAAAAqmO6cYkdjeRNDAJJTBkeiQwQ18di&gclid=Cj0KCQjw2tHABhCiARIsANZzDWoeJMPlqe-lmFAkq48BwtAS7Vb_IAckuwN_1NMsBmMYNoSTmUJs5CIaAqAXEALw_wcB
Europe	Scotland	The University of Edinburgh	Understanding Planetary Health & Data: An Introduction to the Concepts and Themes	Online	Undergraduate and Graduate Course	English	1 semester	$17,595.40	Yes	No	No		http://www.drps.ed.ac.uk/20-21/dpt/cxvesc11243.htm
Europe	Scotland	Unviersity of Edinburgh	PgCert Global Health Challenges	Online	Graduate Course	English	1 year	$8,462.58	Yes	No	No		https://global.ed.ac.uk/msc-global-challenges/programmes/health
Europe	Scotland	University of Edinburgh	Human Health in the Antrhopocene	In Person	Graduate Course	English	1 Semester	$16,271.18	Yes	No	No		http://www.drps.ed.ac.uk/23-24/dpt/cxefie11187.htm
Europe	Scotland	University of Glasgow	Planetary Health MED5019	In Person	Graduate Course	English	1 semester	$21,128.02	Yes	No	No		https://www.gla.ac.uk/coursecatalogue/course/?code=MED5019
Europe	Spain	Universitat Oberta de Catalunya, Universitat Pompeu Fabra, and Institute of Global Health	Masters Degree in Planetary Health	Online	Graduate Program	Spanish, Catalan	1-2 years	$5,438.41	No	No	No		https://www.uoc.edu/ca/estudis/masters/master-universitari-salut-planetaria
Europe	Spain	Pompeu Fabra University	Minor in Planetary Wellbeing	In Person	Undergraduate Program	English, Spanish, Catalan	1 year	$9,598.50	No	No	No		https://www.upf.edu/web/minors/minor-en-benestar-planetari

Region	Country	Institution Name	Course name	Format	Course Type	Available Languages	Length	Costs ($USD)	Is country top 10 most polluting?	Is country top 10 most vulnerable to climate change?	Relationship between planetary health and surgery addressed?	If yes, what:	Link
Europe	Sweden	Linnaeus University	Planetary Health Geography	In Person	Graduate Course	English	5 weeks	$1,709.28	No	No	No		https://planetaryhealthalliance.org/wp-content/uploads/2025/02/LinnaeusUniversity_PlanetaryHealthGeography.pdf (Inactive)
Europe	Switzerland	ETH Zurich	Worldviews - From Sustainability to Regeneration	Online	Open Access Course	English	12 weeks	Free	No	No	No		https://www.edx.org/learn/ethics/eth-zurich-worldviews-from-sustainability-to-regeneration
Europe	Switzerland	Medecins Sans Frontieres/ Tembo	Introduction To Planetary Health - Basic Concepts	Online	Professional Development Course	English	*Missing	Free	No	No	No		https://tembo.msf.org/course/info.php?id=972
Europe	Switzerland	James Lind Institute	MPH in One Health and Planetary Health	Online	Graduate Program	English	1.5-2 years	HIC: $6020 LMIC: $3940.36	No	No	No		https://jliedu.ch/courses/mph-in-one-health-and-planetary-health/
Europe	Switzerland	Geneva Centre of Humanitarian Studies	Planetary Health	Online	Certificate	English	2 weeks	$2,057.64	No	No	No		https://humanitarianstudies.ch/education/planetary-health/#:~:text=Planetary%20health%20focuses%20on%20the,on%20ecosystems%20and%20human%20health.
Europe	Switzerland	The University of Geneva, Institute Pasteur, University of Montreal and Centre Virchow-Villerme/University Paris Descartes	Global Health at the Human Animal Ecosystem Interface	Online	Open Access Course	English	8 weeks	Free	No	No	No		https://www.coursera.org/learn/global-health-human-animal-ecosystem
Europe	Switzerland	Universite de Geneve	(Short Course) Planetary Health	Online	Professional Development Course	English	2 weeks	$2,057.64	No	No	No		https://www.unige.ch/formcont/en/courses/planetary-health
Europe	Switzerland	University of Applied Sciences Northwestern Switzerland	CAS Health and Environment	Hybrid	Certificate	German	18 days	$6,987.94	No	No	No		https://www.fhnw.ch/de/weiterbildung/lifesciences/cas-gesundheit-und-umwelt
Europe	Switzerland	University of Geneva	SDG Summer School: Planetary Health	In Person	Certificate	English	12 days	$363.11 - $2057.64	No	No	No		https://www.unige.ch/genevasummerschools/programme/courses/sdg-summer-school
Europe	Switzerland	Lugano Summer School in Public Health	Planetary Health Action	In Person	Certificate	English	3 days	$1,210.38	No	No	No		https://www.ssph-lugano-summerschool.ch/courses/planetary-health-action
Europe	UK	University of Cambridge	Planetary and Human Health	In Person	Graduate Course	English	4 days	$26,133.75	Yes	No	No		https://www.phpc.cam.ac.uk/education-and-training/postgraduate-teaching/planetary-and-human-health
Europe	England	University of Lincoln	MSc Environment and Planetary Health	In Person	Graduate Program	English	1 year	$20,515.10	Yes	No	No		https://www.lincoln.ac.uk/course/envplhms/
Global		UNCC: e-learn	Human Health and Climate Change: e-course	Online	Certificate	English, Spanish, French	2 hours	Free	N/A	N/A	No		https://www.who.int/publications/m/item/human-health-and-climate-change--e-course
Latin America	Brazil	Universidade de Sao Paulo	Health and Climate Change (Saúde e Mudanças Climáticas)	In Person	Undergraduate Course	Portuguese	6 weeks	Free	Yes	No	No		https://uspdigital.usp.br/janus/componente/disciplinasOferecidasInicial.jsf?action=3&sgldis=MPR5779
Latin America	Brazil	TelessaudesRS-UFRGS	Planetary Health for Primary Care	Online	Open Access Course	English	7-8 weeks	Free	Yes	No	No		https://www.ufrgs.br/telessauders/saude-planetaria/#introducao
Latin America	Brazil	TelessaudesRS-UFRGS	Planetary Health	Online	Open Access Course	English and Portugese	8 weeks	Free	Yes	No	No		https://www.ufrgs.br/telessauders/saude-planetaria/#introducao
Latin America	Chile	Pontif. Universdad Catolica de Chile	Salud Planetaria & Cambio Social: Estrategias para la Acción	In Person	Undergraduate Course	Spanish	1 Semester	*Missing	No	No	No		https://formaciongeneral.uc.cl/explora-los-cursos/salud-planetaria-cambio-social-estrategias-para-la-accion/
Latin America	Chile	Sociedad Chilena de Salud Planetaria	Curso en espanol de Salud Planetaria	Online	Certificate	Spanish	11 hours	Free	No	No	No		https://www.sochisap.org/actividades/curso-salud-planetaria
Latin America	Mexico	Escuela de Salud Punlica de Mexico	Salud planetaria: nuevo enfoque de salud pública globa	Online	Certificate	Spanish	30 hours	Free	No	No	No		https://educacioncontinua.espm.mx/paspe/detalle/programa-salud-planetaria-nuevo-enfoque-de-salud-publica-global-223
North America	Canada	University of Toronto	Planetary Health	Online	Graduate Course	English	1 semester	$11,014.37	Yes	No	No		https://www.dlsph.utoronto.ca/course/planetary-health/
North America	Canada	University of Toronto	Taking Action on Planetary Health: Building community to advance planetary health	Online	Professional Development Course	English	13 weeks	$637.34	Yes	No	No		https://planetaryhealthaction.ca/
North America	Canada	University of Victoria	Transformative Governance for Planetary Health	Online	Graduate Course	English	1 semester	$1,611.84	Yes	No	No		https://planetaryhealthalliance.org/wp-content/uploads/2025/02/UniversityofVictoria_TransformativeGovernanceforPlanetaryHealth.pdf (Inactive)
North America	Canada	Quebec's National Institute of Public Health	Changements climatiques et sante: prevenir, soigner et s'adapter	Online	Open Access Course	French	10 hours	Free	Yes	No	No		https://www.fun-mooc.fr/fr/cours/changements-climatiques-et-sante-prevenir-soigner-et-sadapter/
North America	Canada	University of Toronto	Planetary and Global Health Ethics	Online	Graduate Course	English	1 semester	$11,014.37	Yes	No	No		https://www.dlsph.utoronto.ca/course/planetary-and-global-health-ethics/
North America	Canada	University of Alberta	Climate Change and Human Health	In Person	Graduate Course	English	1 semester	$6,224.36	Yes	No	No		https://apps.ualberta.ca/catalogue/course/sph/556
North America	Canada	CoPEH-Canada	Hybrid Course on Ecosystem Approaches to Health	Hybrid	Certificate	English	1 month	$35-$430, Varies based on student or professional, HIC or LMIC	Yes	No	No		https://copeh-canada.org/en/key-areas/training-and-capacity-building/course.html
North America	Canada	CASCADES	Introduction to Sustainable Health Systems	Online	Open Access Course	English	1 hour	Free	Yes	No	No		https://cascadescanada.ca/training/asynchronouscourse/

Region	Country	Institution Name	Course name	Format	Course Type	Available Languages	Length	Costs ($USD)	Is country top 10 most polluting?	Is country top 10 most vulnerable to climate change?	Relationship between planetary health and surgery addressed?	If yes, what:	Link
North America	Canada	CASCADES	Fundamentals of sustainable health systems	Online	Open Access Course	English	4 hours	Free	Yes	No	No		https://cascadescanada.ca/training/fundamentals/
North America	Canada	CASCADES	Applied fundamentals of sustainable health systems: Sustainable perioperative care	Online	Open Access Course	English	5 hours	Free	Yes	No	Yes	Aim is to create advocates for a bottom-up and top-down shift toward more environmentally sustainable perioperative care.	https://cascadescanada.ca/training/applied-fundamentals/
North America	Canada	CASCADES	Climate Conscious Inhalers Prescribing	Online	Open Access Course	English	6 hours	Free	Yes	No	No		https://cascadescanada.ca/event/quebec-focused-climate-conscious-inhaler-prescribing-course/
North America	Canada	CASCADES	Metrics for Change: Towards Sustainable Health Systems	Online	Open Access Course	English	12 hours	Free	Yes	No	No		https://cascadescanada.ca/event/metrics-for-change-towards-sustainable-health-systems-spring2024/
North America	Canada	CASCADES	Environmentally Sustainable Kidney Care Workshop	Online	Open Access Course	English	4 hours	Free	Yes	No	No		https://cascadescanada.ca/event/environmentally-sustainable-kidney-care-workshop-june2024/
North America	Canada	CASCADES	Leadership for Change: Toward Sustainable Health Systems Course	Online	Open Access Course	English	4 days	Free	Yes	No	No		https://cascadescanada.ca/training/leadership/#:~:text=Course%20Description,profound%20environmental%20and%20sustainability%20challenges.
North America	Canada	CASCADES	Summer Institute on Sustainable Health Systems	Hybrid	Open Access Course	English	4 days	Free	Yes	No	No		https://cascadescanada.ca/training/summer-institute/
North America	Canada	CASCADES	Using QI to build sustainable, low carbon, resilient and equitable healthcare: Collaboration with CQUIPS+	Online	Certificate	English	4 hours	$289.40	Yes	No	No		https://cascadescanada.ca/training/using-qi-to-build-sustainable-low-carbon-resilient-and-equitable-healthcare/
North America	Canada	CASCADES	Leading sustainable health systems: Collaboration with Canadian Medical Association	Online	Certificate	English	4 weeks	$1,012.91	Yes	No	No		https://cascadescanada.ca/event/leading-sustainable-health-systems-may-11-june-1-2022/
North America	Canada	CASCADES	Leadership Sprint: Climate resilient, low carbon sustainable health systems	Online	Open Access Course	English	4 days	Free	Yes	No	No		https://cascadescanada.ca/event/cascades-leadership-sprint-climate-resilient-low-carbon-sustainable-health-systems/
North America	Canada	University of Alberta	Certifcate in Planetary Health	In Person	Undergraduate Course	English	1 Semester	$2,527.14	Yes	No	No		https://www.ualberta.ca/en/admissions-programs/embedded-undergraduate-certificates/planetary-health.html
North America	Canada	University of British Columbia	Planetary Health / Human Health	In Person	Undergraduate Course	English	1 Semester	$6,142.26	Yes	No	No		https://okanagan.calendar.ubc.ca/course-descriptions/courses/hinto-321-planetary-health-human-health
North America	USA	Yale University	Climate change and health certificate	Online	Professional Development Course	English	18 weeks	$2,000.00	Yes	No	No		https://ysph.yale.edu/cchcert/
North America	USA	Harvard University	The Health effects of climate change	Online	Open Access Course	English	7 weeks	Free	Yes	No	No		https://pll.harvard.edu/course/health-effects-climate-change
North America	USA	Stanford University	Global Leaders and Innovators in Human and Planetary Health	In Person	Undergraduate and Graduate Course	English	1 semester	$21,709.00	Yes	No	No		https://hph.stanford.edu/education/hph-courses
North America	USA	Stanford University	Sustainable Adaptation	In Person	Undergraduate Course	English	1 semester	$21,709.00	Yes	No	No		https://hph.stanford.edu/education/hph-courses
North America	USA	Stanford University	Renewable Energy Transition in Rural America, a Human & Planetary Health Action Lab	In Person	Graduate Course	English	1 semester	$21,709.00	Yes	No	No		https://hph.stanford.edu/education/hph-courses
North America	USA	Stanford University	Climate Change, Toxins, and Health: From Education to Action	In Person	Undergraduate and Graduate Course	English	1 semester	$21,709.00	Yes	No	No		https://hph.stanford.edu/education/hph-courses
North America	USA	Stanford University	Contemplative Competence for Sustainability of Public and Planetary Health and Well-being	In Person	Undergraduate and Graduate Course	English	1 semester	$21,709.00	Yes	No	No		https://hph.stanford.edu/education/hph-courses
North America	USA	Stanford University	Global Change and Emerging Infectious Disease	In Person	Undergraduate and Graduate Course	English	1 semester	$21,709.00	Yes	No	No		https://hph.stanford.edu/education/hph-courses
North America	USA	Stanford University	Health Policy Analysis and Population Health	In Person	Undergraduate and Graduate Course	English	1 semester	$21,709.00	Yes	No	No		https://hph.stanford.edu/education/hph-courses
North America	USA	Stanford University	Geography of Health	In Person	Undergraduate and Graduate Course	English	1 semester	$21,709.00	Yes	No	No		https://hph.stanford.edu/education/hph-courses
North America	USA	Downstate Health Sciences University	Advanced Certificate Program in Climate Change and Planetary Health	Online	Certificate	English	2.5 years	$61,250.00	Yes	No	No		https://www.downstate.edu/education-training/school-of-public-health/programs/advanced-climate-change.html
North America	USA	University of Minnesota	Social Determinants of Health: Planetary Health	Online	Open Access Course	English	2 months	Free	Yes	No	No		https://www.coursera.org/specializations/social-determinants-of-health-data-to-action

Region	Country	Institution Name	Course name	Format	Course Type	Available Languages	Length	Costs ($USD)	Is country top 10 most polluting?	Is country top 10 most vulnerable to climate change?	Relationship between planetary health and surgery addressed?	If yes, what:	Link
North America	USA	University of Minnesota	Planetary Health and Global Climate Change: A Whole Systems Healing Approach	In Person	Undergraduate and Graduate Course	English	1 semester	$19,259.00	Yes	No	No		https://planetaryhealthalliance.org/wp-content/uploads/2025/02/UniversityOfMinnesota_PlanetaryHealthandGlobalClimateChange.pdf
North America	USA	Harvard T.H. Chan School of Public Health	An Introduction to Planetary Health	In Person	Undergraduate and Graduate Course	English	1 semester	$29,660.00	Yes	No	No		https://salatainstitute.harvard.edu/courses/an-introduction-to-planetary-health/
North America	USA	George Mason University	Planetary Health	Online	Undergraduate and Graduate Course	English	1 semester	$19,344.00	Yes	No	No		https://science.gmu.edu/media/evpp-428-evpp-528-planetary-health-spring-2023-bayles
North America	USA	Child Family Health International (NGO)	Virtual Global Health Elective	Online	Graduate Course	English	4 weeks	HIC: $495 LMIC: $250	Yes	No	No		https://www.cfhi.org/program/virtual-global-health-elective/
North America	USA	Hutton Honors College: Indiana University Bloomington	Planetary Health and Global Climate Change in Costa Rica	In Person	Undergraduate Course	English	3 weeks	$4,000.00	Yes	No	No		https://hiep.indiana.edu/hhsa/upcoming-programs/costa-rica.html
North America	USA	City University of New York GRADUATE SCHOOL OF PUBLIC HEALTH & HEALTH POLICY	PhD degree in Environmental and Planetary Health Sciences	In Person	Graduate Program	English	5 years	$6,910,800.00	Yes	No	No		https://sph.cuny.edu/academics/degrees-and-programs/doctoral-programs/phd-in-environmental-and-planetary-health-sciences/
North America	USA	Cornell University	Planetary Health	In Person	Graduate Course	English	1 semester	$3,557.00	Yes	No	No		https://publichealth.cornell.edu/curriculum/coursework/
North America	USA	University of North Carolina School of Medicine	Planetary Health Scholarly Concentration	In Person	Medical School	English	4 years	$63,334.00	Yes	No	No		https://www.med.unc.edu/md/curriculum/opportunities-and-programs/scholarly-concentrations/
North America	USA	Yale University	Planetary Health for Nurses	Online	Open Access Course	English primarily Auto-translated to Dutch, Arabic, Portugese, Bahasa Indonesia, French, Spanish, Thai	17 hours	Free	Yes	No	No		https://online.yale.edu/courses/planetary-health-nurses
North America	USA	Brown University	Planetary Health: Global Environmental Change and Emeging Infectious Disease	Online	Graduate Course	English	1 semester	$8,962.00	Yes	No	No		https://graduatestudies.biomed.brown.edu/sites/default/files/Brown_Pfizer_PlanetaryHealth_Syllabus_2025.pdf
North America	USA	Dominican University of California	Minor in Planetary Health	In Person	Undergraduate Program	English	4 years	$211,420.00	Yes	No	No		https://dominican.smartcatalogiq.com/en/2024-2025/academic-catalog/undergraduate-degree-programs/global-public-health/planetary-health-minor/
North America	USA	University of California, Los Angeles	Planetary Health: Consequences of Environmental Change for Human Health	In Person	Undergraduate Course	English	1 semester	$5,277.13	Yes	No	No		https://catalog.registrar.ucla.edu/course/2023/envhlt216?siteYear=2023
North America	USA	The University of Arizona	MPH One Health	In Person	Graduate Program	English	2-4 years	$84,765.66	Yes	No	No		https://publichealth.arizona.edu/programs/graduate/mph/one-health
North America	USA	Columbia University	European Climate and Health Responders Course	Online	Open Access Course	English	2 months	Free	Yes	No	No		https://www.publichealth.columbia.edu/research/programs/global-consortium-climate-health-education/courses/past-courses/european-climate-health-responder-course
North America	USA	University of California Davis	Rx One Health	In Person	Certificate	English	2 weeks	$3,500.00	Yes	No	No		https://rxonehealth.vetmed.ucdavis.edu/
North America	USA	University of Colorado	Diploma in Climate Medicine	Hybrid	Professional Development Course	English	5 weeks	S2900 for trainees and health professionals $3700 for physicians	Yes	No	No		https://medschool.cuanschutz.edu/climateandhealth/diploma-in-climate-medicine#ft-certificate-descriptions-2
North America	USA	University of New Mexico	A Systems Approach to Planetary Health	Hybrid	Undergraduate and Graduate Course	English	1 Semester	$17,064.50	Yes	No	No		https://planetaryhealthalliance.org/wp-content/uploads/2025/02/UniversityOfNewMexico_ASystemsApproachToPlanetaryHealth.pdf
North America	USA	Stanford University	PhD in Environment and Resources	In Person	Graduate Program	English	4-5 years	Free	Yes	No	No		https://eiper.stanford.edu/academics-admissions/phd-degree
North America	USA	Stanford University	Planetary Health Postdoctoral Fellowship	In Person	Postdoctoral Fellowship	English	2-4 years	Free	Yes	No	No		https://globalhealth.stanford.edu/programs/post-doctoral-fellowship-in-planetary-health/
North America	USA	Duke University	Planetary Health: Ecosystems, Human Health and Policy	In Person	Undergraduate Course	English	1 semester	$35,132.50	Yes	No	No		https://trinity.duke.edu/courses/fall-2021/planetary-health-ecosystems-human-health-and-policy
North America	USA	Emory University	Introduction to Environmental Health	In Person or Online	Graduate Course	English	1 semester	$25,093.00	Yes	No	No		https://sph.emory.edu/academics/courses/eh-courses/index.html
North America	USA	Emory University	Certificate in Climate and Health	In Person	Certificate	English	1 semester	$25,093.00	Yes	No	No		https://sph.emory.edu/academics/certificates/climate-health/index.html
North America	USA	Harvard University	The Role of Soil Health In creating Sustainable Food Systems	Online	Undergraduate and Graduate Course	English	1 semester	$30,838.00	Yes	No	No		https://harvard.simplesyllabus.com/api2/doc-pdf/8krkhewzk/Spring-Term-2024-Full-Term-ENVR-E-129c-1-The-Role-of-Soil-Health-in-Creating-Sustainable-Food-Systems.pdf?locale=en-US

Region	Country	Institution Name	Course name	Format	Course Type	Available Languages	Length	Costs ($USD)	Is country top 10 most polluting?	Is country top 10 most vulnerable to climate change?	Relationship between planetary health and surgery addressed?	If yes, what:	Link
North America	USA	McDaniel College	Health in Anthropocene	Hybrid	Undergraduate Course	English	1 Semester	$5,674.00	Yes	No	No		https://selfservice.campus.mcdaniel.edu:8173/Student/Courses/Search?subjects=ENV
North America	USA	Northwestern University	Planetary Health: Health in the Anthropocene	Online	Graduate Course	English	1 Semester	$13,240.00	Yes	No	No		https://class-descriptions.northwestern.edu/4990/UC/MSGH/42485
North America	USA	Ohio State University	Sustainable Agriculture and Food Systems	In Person	Undergraduate and Graduate Course	English	1 Semester	$6,090.00	Yes	No	No		https://senr.osu.edu/courses/enr-5600
North America	USA	University of Miami	Urban Environment and Public Health	In Person	Graduate Course	English	1 Semester	$23,435.00	Yes	No	No		https://bulletin.miami.edu/courses-az/eph/
North America	USA	University of Miami	Environmental Health	In Person	Graduate Course	English	1 Semester	$23,435.00	Yes	No	No		https://bulletin.miami.edu/courses-az/eph/
North America	USA	University of Miami	Climate and Health	In Person	Graduate Course	English	1 Semester	$23,435.00	Yes	No	No		https://bulletin.miami.edu/courses-az/eph/
North America	USA	University of Miami	Toxicology: Climate and Health	In Person	Graduate Course	English	1 Semester	$23,435.00	Yes	No	No		https://bulletin.miami.edu/courses-az/eph/
North America	USA	University of Miami	Climate, Environment, and Health: Data Integration and Management	In Person	Graduate Course	English	1 Semester	$23,435.00	Yes	No	No		https://bulletin.miami.edu/courses-az/eph/
North America	USA	University of Miami	Climate, Cool Cities, Healthy Communities	In Person	Graduate Course	English	1 Semester	$23,435.00	Yes	No	No		https://bulletin.miami.edu/courses-az/eph/
North America	USA	University of Miami	Ecology and Control of Vector-Borne Diseases	In Person	Graduate Course	English	1 Semester	$23,435.00	Yes	No	No		https://bulletin.miami.edu/courses-az/eph/
North America	USA	University of Miami	Public Health Seminar	In Person	Graduate Course	English	1 Semester	$23,435.00	Yes	No	No		https://bulletin.miami.edu/courses-az/eph/
North America	USA	University of Miami	Public Health Nutrition	In Person	Graduate Course	English	1 Semester	$23,435.00	Yes	No	No		https://bulletin.miami.edu/courses-az/eph/
North America	USA	University of Miami	Analysis of the Health Effects of Climate	In Person	Graduate Course	English	1 Semester	$23,435.00	Yes	No	No		https://bulletin.miami.edu/courses-az/eph/
North America	USA	University of Vermont	Foods for Planetary Health	In Person	Undergraduate Course	English	1 Semester	$22,323.00	Yes	No	No		https://catalogue.uvm.edu/undergraduate/courses/courselist/nfs/
North America	USA	University of Wisconsin	Air Pollution and Human Health	In Person	Undergraduate Course	English	1 Semester	$21,052.00	Yes	No	No		https://nelson.wisc.edu/undergraduate/featured-courses/air-pollution-and-human-health/
North America	USA	University of Wisconsin	Climate Change, Human and Planetary Health	In Person	Graduate Course	English	1 Semester	$12,826.00	Yes	No	No		https://pophealth.wisc.edu/degree-programs/courses-and-syllabi/
North America	USA	University of Wisconsin	Introduction to Environmental Health	In Person	Undergraduate Course	English	1 Semester	$21,052.00	Yes	No	No		https://nelson.wisc.edu/undergraduate/featured-courses/introduction-to-environmental-health/
North America	USA	Cornell University	Health, Wellbeing, & the Environment	In Person	Graduate Course	English	1 Semester	$22,384.00	Yes	No	No		https://publichealth.cornell.edu/curriculum/coursework/
North America	USA	Cornell University	Leading Change for Health Equity, Sustainability, & Justice	In Person	Graduate Course	English	1 Semester	$22,384.00	Yes	No	No		https://publichealth.cornell.edu/curriculum/coursework/
North America	USA	Cornell University	Public Health & Emergency Management	In Person	Graduate Course	English	1 Semester	$22,384.00	Yes	No	No		https://publichealth.cornell.edu/curriculum/coursework/
North America	USA	George Mason University	Conservation Medicine	Online	Undergraduate and Graduate Course	English	1 Semester	$19,344.00	Yes	No	No		https://catalog.gmu.edu/search/?P=EVPP%20527
North America	USA	University of Arizona	Environmental and Occupational Health	In Person	Graduate Course	English	1 Semester	$11,700.00	Yes	No	No		https://hub.coph.arizona.edu/sites/default/files/courses/EHS%20575%20Course%20Information.pdf
North America	USA	University of Arizona	Global to Local: Environmental Change and Human Health	In Person	Undergraduate and Graduate Course	English	1 Semester	$21,550.00	Yes	No	No		https://geography.arizona.edu/course/ehs-525-global-local-environmental-change-and-human-health
North America	USA	University of Arizona	One Health Foundations	In Person	Undergraduate and Graduate Course	English	1 Semester	$21,550.00	Yes	No	No		https://hub.coph.arizona.edu/sites/default/files/courses/EHS%20545%20Course%20Information.pdf
North America	USA	University of Arizona	Climate and Health	In Person	Undergraduate and Graduate Course	English	1 Semester	$21,550.00	Yes	No	No		https://catalog.arizona.edu/courses?page=1&cq=
North America	USA	University of Arizona	Food in 2050 and Beyond: Climate Change and Global Health	In Person	Undergraduate and Graduate Course	English	1 Semester	$21,550.00	Yes	No	No		https://catalog.arizona.edu/courses?page=1&cq=
North America	USA	University of California Berkeley	Introduction to Environmental Health Sciences	In Person	Graduate Course	English	1 Semester	$6,381.00	Yes	No	No		https://guide.berkeley.edu/courses/pb_hlth/
North America	USA	University of California Berkeley	Health Risk Assessment	In Person	Graduate Course	English	1 Semester	$6,381.00	Yes	No	No		https://classes.berkeley.edu/content/2020-spring-pbhlth-220c-001-lec-001
North America	USA	University of California Berkeley	Environmental Determinants of Infectious Disease	In Person	Graduate Course	English	1 Semester	$6,381.00	Yes	No	No		https://classes.berkeley.edu/content/2018-Fall-PBHLTH-273-001-LEC-001
North America	USA	University of California Berkeley	Health Implications of Climate Change	In Person	Graduate Course	English	1 Semester	$6,381.00	Yes	No	No		https://classes.berkeley.edu/content/2022-spring-pbhlth-271g-001-lec-001
North America	USA	University of San Diego	Engineering and the Health of the Planet	Online	Graduate Course	English	1 Semester	$10,920.00	Yes	No	No		https://onlinedegrees.sandiego.edu/masters-engineering-sustainability-health/classes/
North America	USA	University of San Diego	Health and Built Environment	Online	Graduate Course	English	1 Semester	$10,920.00	Yes	No	No		https://onlinedegrees.sandiego.edu/masters-engineering-sustainability-health/classes/

Region	Country	Institution Name	Course name	Format	Course Type	Available Languages	Length	Costs ($USD)	Is country top 10 most polluting?	Is country top 10 most vulnerable to climate change?	Relationship between planetary health and surgery addressed?	If yes, what:	Link
North America	USA	Emory University	Climate Crisis and Clinical Medicine Virutal Elective for Medical Students	Online	Medical School	English	4 weeks	$222,800.00	Yes	No	Yes	Discusses how to "green" the operating room and how to reduce its climate impact.	https://medbox.org/document/climate-crisis-and-clinical-medicine-virtual-elective-for-medical-students-online-course<br><br>https://sustainability.emory.edu/climate-crisis-and-clinical-medicine-virtual-elective-for-medical-students/
North America	USA	University of Michigan	Act on Climate: Steps to Individual, Community and Political Action	Online	Open Access Course	English	7 weeks	Free	Yes	No	No		https://online.umich.edu/courses/act-on-climate-steps-to-individual-community-and-political-action/
North America	USA	Project Drawdown	Climate Solutions 101, Presented by Project Drawdown	Online	Open Access Course	English	6 hours	Free	Yes	No	No		https://drawdown.org/climate-solutions-101
North America	USA	Harvard University	Climate Change, Planetary Health, and Medicine	Online	Professional Development Course	English	2 days	$1250-$1500, Variable based on profession	Yes	No	No		https://cmecatalog.hms.harvard.edu/climate-change-planetary-health-medicine (Inactive)
North America	USA	Brown University	Scholarly Concentration in Planetary Health	In Person	Medical School	English	4 years	$292,600.00	Yes	No	No		https://education.med.brown.edu/scholarly-concentrations-program/planetary-health
North America	USA	Massachusetts Institute of Technology	D-Lab: Climate Change and Planetary Health	In Person	Undergraduate and Graduate Course	English	1 Semester	15 497.5	Yes	No	No		https://d-lab.mit.edu/academics/classes/d-lab-water-climate-change-health
North America	USA	Harvard University	Climate Change and Planetary Health	In Person	Graduate Program	English	1 year	$74,340.00	Yes	No	No		https://hsph.harvard.edu/degrees-and-programs/concentrations/climate-planetary-health/
North America	USA	University of Minnesota	CSPH 5644: Nature Rx: Planetary Health, Plants, and People	Online	Undergraduate and Graduate Course	English	1 semester	$9,074.00	Yes	No	No		https://csh.umn.edu/academics/for-credit-courses/csph-5644-nature-rx-planetary-health-plants-and-people
North America	USA	University of Vermont	Planetary Health and Agroecology	In Person	Graduate Course	English	1 semester	$5,580.00	Yes	No	No		https://www.uvm.edu/instituteforagroecology/planetary-health-and-agroecology
North America	USA	University of Pennsylvania	Planetary Health and Oral Health	In Person	Graduate Course	English	1 Semester	$47,088.00	Yes	No	No		https://environment.upenn.edu/resources/courses?combine=planetary%20health&field_nc_topics_target_id=All&field_nc_departments_target_id=All&field_nc_priority_target_id=All
North America	USA	The Alliance of Nurses for Healthy Environments (ANHE)	Plastic Pollution and the Impact on Human Health	Online	Certificate	English	1 hour	Free	Yes	No	No		https://learning.envirn.org/enrol/index.php?id=124
North America	USA	The Alliance of Nurses for Healthy Environments (ANHE)	Climate Readiness: Preparation is Key	Online	Certificate	English	1 hour	Free	Yes	No	No		https://learning.envirn.org/enrol/index.php?id=111
North America	USA	The Alliance of Nurses for Healthy Environments (ANHE)	The Disposal of Medical Waste in West Africa: An Environmental and Occupational	Online	Certificate	English	1 hour	Free	Yes	No	No		https://learning.envirn.org/course/view.php?id=118
North America	USA	The Alliance of Nurses for Healthy Environments (ANHE)	Nursing on the Frontlines of the Climate Crisis: Education for Action	Online	Certificate	English	10 hours	Free	Yes	No	No		https://learning.envirn.org/course/index.php?categoryid=40
North America	USA	Portland State University	MPH in Environmental Systems & Human Health	In Person	Graduate Program	English	2-3 years	$45,720.00	Yes	No	No		https://ohsu-psu-sph.org/degree-programs/master-public-health/mph-eshh/
Oceania	Australia	Victoria University	Undergraduate Certifcate in Science and the Environment	In Person	Certificate	English	0.5 years	$9,059.74	No	No	No		https://www.vu.edu.au/courses/undergraduate-certificate-in-science-and-the-environment-nuse
Oceania	Australia	Monash University	Master of Public Health (Global and Planetary Health)	In Person	Graduate Program	English	1-2 years	$33,200.47	No	No	No		https://www.monash.edu/study/courses/find-a-course/public-health-m6024?international=true
Oceania	Australia	Victoria University	Master of Global Public Health	In Person	Graduate Program	English	2 years	$40,452.67	No	No	No		https://www.vu.edu.au/courses/master-of-global-public-health-hmgp#fees-and-scholarships
Oceania	Australia	Deakin University	Promoting Human and Planetary Health	Online	Graduate Course	English	6 weeks	$887.60	Yes	No	No		https://www.deakin.edu.au/study/find-a-course/short-courses/stackable-short-courses/promoting-human-and-planetary-health
Oceania	Australia	Monash University	Sustainable Healthcare in Practice	Online	Professional Development Course	English	11 weeks	$1,936.59	No	No	No		https://www.monash.edu/msdi/study/professional-development/sustainable-healthcare-in-practice
Oceania	Australia	The University of Melbourne	Planetary and Global Health	Hybrid	Graduate Course	English	2 weeks	$4,921.52	No	No	No		https://handbook.unimelb.edu.au/2022/subjects/poph90230
Oceania	Australia	The University of Sydney	Food, Sustainability and Planetary Health	In Person	Undergraduate Course	English	1 semester	$17,300.21	No	No	No		https://www.sydney.edu.au/units/EDGU1007
Oceania	Australia	Australian National University	Planetary Health Equity Future Leaders Program 2025	In Person	Professional Development Course	English	12 days	Free	No	No	No		https://hothouse.anu.edu.au/event/planetary-health-equity-future-leaders-program-2025
Oceania	Australia	Monash University	Sustainable Healthcare Fundamentals	In Person	Professional Development Course	English	20 hours	$631.63	No	No	No		https://www.monash.edu/msdi/study/professional-development/sustainable-healthcare-fundamentals
Oceania	New Zealand	Auckland University	Public and Environmental Health Major (Bachelor of Health Science)	In Person	Undergraduate Program	English	3 years	$145,374.18	No	No	No		https://www.aut.ac.nz/study/study-options/health-sciences/courses/bachelor-of-health-science/public-and-environmental-health-major
Europe	Greece	Aristotle University	Environment and Health	In Person	Medical School	English	6 years	$81,853.20	No	No	No		https://qa.auth.gr/en/class/1/200011418
Asia	India	St. John's Medical College	'Citizen Doctor	In Person	Medical School	English	5.5 years	$50,910.64	Yes	No	No		https://stjohns.in/medicalcollege/Citizen_Doctor_Course.php

Region	Country	Institution Name	Course name	Format	Course Type	Available Languages	Length	Costs ($USD)	Is country top 10 most polluting?	Is country top 10 most vulnerable to climate change?	Relationship between planetary health and surgery addressed?	If yes, what:	Link
Europe	Switzerland	Universite de Lausanne	When the Earth hurts us and vice versa: impact of pollution on human health	In Person	Medical School	French	6 years	$8,477.28	No	No	No		https://www.unil.ch/ecoledemedecine/cours-option-bmed3
Asia	Turkey	Cukurova Universitesi	Sustainable Health Service Delivery	In Person	Undergraduate Course	Turkish	1 Semester	$118.07	No	No	No		https://eobs.cu.edu.tr/Ders/GenelBilgi/638639
Asia	Turkey	Ankara University	Planetary Health	In Person	Medical School	Turkish and English	6 years	$110,400.00	No	No	No		https://bbs.ankara.edu.tr/Ders_Bilgileri.aspx?sdgNo=1105571&dno=1869249&bno=4309&bot=1929
North America	Canada	Dalhousie University	Interprofessional Education Mini-Course: Healthcare in a Changing Climate: The Need for Planetary Health & Sustainable Health Systems	Hybrid	Medical School	English	4 years	$72,993.84	Yes	No	No		https://medicine.dal.ca/departments/core-units/global-health/planetaryhealth.html#:~:text=Working%20together%20with%20CASCADES%20and,Summer%20Institute%20on%20Sustainable%20Healthcare
North America	Canada	University of Alberta	Planetary Health	Hybrid	Medical School	English	4 years	$47,000.97	Yes	No	No		https://www.ualberta.ca/en/medicine/programs/md/program/curriculum/electives/year-1-2.html?0=planet&details=planetary-health
North America	Canada	University of Manitoba	Community Health Sciences Electives	In Person	Medical School	English	4 years	$36,399.37	Yes	No	No		https://umanitoba.ca/medicine/undergraduate-medical-education/electives
North America	Canada	University of Ottawa	Concentration in Global Health and Social Accountability	Hybrid	Medical School	English	4 years	$85,914.69	Yes	No	No		https://www.uottawa.ca/faculty-medicine/global-health-learning-opportunities
North America	Canada	University of Saskatchewan	Environmental Medicine	In Person	Medical School	English	4 years	$66,313.84	Yes	No	No		https://medicine.usask.ca/documents/ugme/syllabi/2024-2025/medc-407-electives-2024-2025-10-1.pdf
Europe	Austria	Medical University of Vienna	An Extensive Health Concept	In Person	Medical School	German and English	6 years	$9,914.04	No	No	No		https://campus.meduniwien.ac.at/med.campus/ee/ui/ca2/app/desktop/#/slc.tm.cp/student/courses/369788?$scrollTo=toc_overview
Europe	Germany	Goethe University	Climate Change and Health	Hybrid	Medical School	German	6 years	Free	Yes	No	No		https://www.allgemeinmedizin.uni-frankfurt.de/102815250/Klimawandel_und_Gesundheit#:~:text=Im%20Dezember%202020%20wurde%20am,dessen%20gesundheitliche%20Auswirkungen%20zu%20sensibilisieren.
Europe	Germany	Martin Luter University Halle-Wittenberg	Climate Change and its Consequences for Healthcare - Clinical Elective	In Person	Undergraduate Course	German	1 Semester	Free	Yes	No	No		https://studip.uni-halle.de/dispatch.php/course/details/index/ffa6e00093b99ce4a1e8836667493f88?set_language=de_DE
Europe	Sweden	Karolinska Institutet	Sustainable Health and Development	In Person	Medical School	Swedish and English	5.5 years	$163,652.38	No	No	No		https://education.ki.se/course-and-programme-syllabi/course-syllabus-2XX081
Europe	Switzerland	Università della Svizzera Italiana	Planetary health: developing the tools for effective health advocacy	In Person	Medical School	German and English	6 years	$58,464.24	No	No	No		https://search.usi.ch/en/courses/35272214/o-030-planetary-health-developing-the-tools-for-effective-health-advocacy
North America	USA	University of Minnesota	Advanced Nursing for Public, Population, and Planetary Health	In Person	Graduate Course	English	1 Semester	$7,803.00	Yes	No	No		https://umtc.catalog.prod.coursedog.com/courses/8268911
North America	Canada	Trinity Western University	Planetary & Global Health	In Person	Undergraduate Course	English	1 Semester	$8,714.01	Yes	No	No		https://www.twu.ca/academics/courses?field_program_reference_target_id_reference=30371
North America	USA	Rush University	Environmental Health	In Person	Graduate Course	English	1 Semester	$16,608.00	Yes	No	No		https://www.rushu.rush.edu/college-nursing/programs-admissions/advanced-public-health-nursing-dnp/advanced-public-health-nursing-dnp-curriculum
North America	USA	Baylor College of Medicine	Humans & Planetary Health: An Ecosystem Approach to Medicine	In Person	Medical School	English	4 years	$131,130.00	Yes	No	No		https://cdn.bcm.edu/sites/default/files/2024-07/school-of-medicine-2024-2025-official-catalog.pdf
North America	USA	Columbia University	Introduction to Global and Population Health	In Person	Graduate Course	English	1 Semester	$20,960.00	Yes	No	No		https://www.pgh.cuimc.columbia.edu/file/1128/download?token=Z7eE1ri3
North America	USA	Loyola University Chicago	Climate Change and Human Health	In Person	Medical School	English	4 years	$260,120.00	Yes	No	No		https://www.luc.edu/stritch/regrec/electivecatalog/coursecatalog/centerforcommunityglobalhealth/ccgh250/
North America	USA	Rutgers University	Climate Health and Environmental Sustainability	In Person	Medical School	English	4 years	$274,256.00	Yes	No	No		https://rwjms.rutgers.edu/education/md/distinction-programs#tab=panel-3
North America	USA	Rutgers University	Climate Health and General Environment	Online	Medical School	English	4 years	$274,256.00	Yes	No	No		https://njms.rutgers.edu/education/registrar/2024/2024-2025%20Fourth%20Year%20Electives.pdf
North America	USA	University of Utah	Sustainability, Medicine & Health	Online	Medical School	English	4 years	$350,912.00	Yes	No	No		https://tools.medicine.utah.edu/som.catalog/catalog.year/year/course_year/a65822883b341ecdeb651ff2535b63e1/syllabus/download
North America	USA	Stanford University	The Impact of Climate Change on Human Health	In Person	Medical School	English	4 years	$254,988.00	Yes	No	No		https://explorecourses.stanford.edu/search?view=catalog&filter-coursestatus-
North America	USA	Indiana University	Climate Change and Health	In Person	Medical School	English	4 years	$240,000.00	Yes	No	No		https://medicine.iu.edu/mse/education/electives
North America	USA	New York Medical College	Children's Environmental Health	In Person	Medical School	English	4 years	$252,320.00	Yes	No	No		https://www.nymc.edu/som/education/md/areas-of-concentration/

Region	Country	Institution Name	Course name	Format	Course Type	Available Languages	Length	Costs ($USD)	Is country top 10 most polluting?	Is country top 10 most vulnerable to climate change?	Relationship between planetary health and surgery addressed?	If yes, what:	Link
North America	USA	Rowan University	Climate Health	In Person	Medical School	English	4 years	$297,472.00	Yes	No	No		https://cmsru.rowan.edu/documents/education-documents/scholarly-concentrations/scholarly-concentration-in-climate-health.pdf
North America	USA	Mayo Clinic College of Medicine and Science	Sustainability in Medicine	Online	Medical School	English	4 years	$271,600.00	Yes	No	No		https://college.mayo.edu/academics/mayo-clinic-alix-school-of-medicine/md-program/curriculum/academic-enrichments/academic-enrichment-opportunities/?page=34#:~:text=Sustainability%20in%20Medicine
North America	USA	Creighton University	Planetary Health	In Person	Medical School	English	4 years	$273,536.00	Yes	No	No		https://catalog.creighton.edu/medicine/all-courses/all-courses.pdf
North America	USA	Creighton University	Planetary Health and Sustainability	In Person	Graduate Course	English	1 semester	$6,150.00	Yes	No	No		https://catalog.creighton.edu/medicine/all-courses/all-courses.pdf
North America	USA	Case Western Reserve University	Climate and Health Pathway	In Person	Medical School	English	4 years	$290,104.00	Yes	No	No		https://case.edu/medicine/md/academics/pathways-programs
North America	USA	Icahn School of Medicine at Mount Sinai	Pediatric Environmental Health Electives	In Person	Medical School	English	4 years	$296,832.00	Yes	No	No		https://icahn.mssm.edu/about/departments/environmental-medicine/education
North America	USA	University of Colorado	Climate Change and Environment	In Person	Medical School	English	4 years	$333,160.00	Yes	No	No		https://ucdenver.oasisscheduling.com/public/courses/index.html?yid=2025&slid=3
North America	USA	University of Colorado	Climate Change & One Health	In Person	Medical School	English	4 years	$333,160.00	Yes	No	No		https://ucdenver.oasisscheduling.com/public/courses/index.html?yid=2025&slid=3
North America	USA	University of Colorado	Environmental Health for Future	In Person	Medical School	English	4 years	$333,160.00	Yes	No	No		https://ucdenver.oasisscheduling.com/public/view_course.html?yid=2025;did=IDPT;cid=8103
North America	USA	University of Maryland	Climate Change, Health, and Society: An Interprofessional Elective	In Person	Medical School	English	4 years	$272,996.00	Yes	No	No		https://www.medschool.umaryland.edu/ome/curriculum/pre-clerkship-curriculum/pre-clerkship-electives/
North America	USA	West Virginia University	Climate Change and Healthcare	Online	Medical School	English	4 years	$279,738.00	Yes	No	No		https://medicine.hsc.wvu.edu/ms4catalog/morgantown-rotations/online-courses/climate-change-and-healthcare-virtual/
North America	USA	University of Nebraska	Climate Change and Health	In Person	Medical School	English	4 years	$206,472.00	Yes	No	No		https://www.unmc.edu/com/education/enrichment/approved-tracks.html
North America	USA	University of Cincinnati	Planetary Health and Medicine	Hybrid	Medical School	English	4 years	$272,350.00	Yes	No	No		https://med.uc.edu/institutes/integrative/education/courses
North America	USA	University of California, Davis	Climate Change: Implications for Clinical Practice & Population Health	In Person	Medical School	English	4 years	$187,572.00	Yes	No	No		https://catalog.ucdavis.edu/courses-subject-code/imd/
North America	USA	University of California, San Francisco	Climate Justice, Environment, Health & Professional Activism	In Person	Medical School	English	4 years	$225,692.00	Yes	No	No		https://coursecatalog.ucsf.edu/course/3830 (Inactive)
